# Clustering of Tir during enteropathogenic *E*. *coli* infection triggers calcium influx–dependent pyroptosis in intestinal epithelial cells

**DOI:** 10.1371/journal.pbio.3000986

**Published:** 2020-12-30

**Authors:** Qiyun Zhong, Theodoros I. Roumeliotis, Zuza Kozik, Massiel Cepeda-Molero, Luis Ángel Fernández, Avinash R. Shenoy, Chris Bakal, Gad Frankel, Jyoti S. Choudhary

**Affiliations:** 1 Centre for Molecular Bacteriology and Infection, Department of Life Sciences, Imperial College, London, United Kingdom; 2 Functional Proteomics Group, Chester Beatty Laboratories, The Institute of Cancer Research, London, United Kingdom; 3 Department of Microbial Biotechnology, Centro Nacional de Biotecnología, Consejo Superior de Investigaciones Científicas (CNB-CSIC), Campus UAM-Cantoblanco, Madrid, Spain; 4 Centre for Molecular Bacteriology & Infection, Department of Infectious Disease, Imperial College, London, United Kingdom; 5 Dynamical Cell Systems, Chester Beatty Laboratories, The Institute of Cancer Research, London, United Kingdom; University of Bern, SWITZERLAND

## Abstract

Clustering of the enteropathogenic *Escherichia coli* (EPEC) type III secretion system (T3SS) effector translocated intimin receptor (Tir) by intimin leads to actin polymerisation and pyroptotic cell death in macrophages. The effect of Tir clustering on the viability of EPEC-infected intestinal epithelial cells (IECs) is unknown. We show that EPEC induces pyroptosis in IECs in a Tir-dependent but actin polymerisation-independent manner, which was enhanced by priming with interferon gamma (IFNγ). Mechanistically, Tir clustering triggers rapid Ca^2+^ influx, which induces lipopolysaccharide (LPS) internalisation, followed by activation of caspase-4 and pyroptosis. Knockdown of caspase-4 or gasdermin D (GSDMD), translocation of NleF, which blocks caspase-4 or chelation of extracellular Ca^2+^, inhibited EPEC-induced cell death. IEC lines with low endogenous abundance of GSDMD were resistant to Tir-induced cell death. Conversely, ATP-induced extracellular Ca^2+^ influx enhanced cell death, which confirmed the key regulatory role of Ca^2+^ in EPEC-induced pyroptosis. We reveal a novel mechanism through which infection with an extracellular pathogen leads to pyroptosis in IECs.

## Abstract 2

This study reveals a novel mechanism by which infection with enteropathogenic E. coli (EPEC) leads to pyroptosis in intestinal epithelial cells; this process is dependent on the type III secretion system effector Tir, and enhanced by priming with IFNγ. Intimin-mediated Tir clustering induces rapid calcium influx, which leads to internalisation of lipopolysaccharide, activation of caspase-4 and cleavage of gasdermin D.

## Introduction

The extracellular pathogen enteropathogenic *Escherichia coli* (EPEC) causes persistent infantile diarrhoea [1] and has been found to preferentially colonise colorectal cancer tissue in adult patients [2]. EPEC infection of the intestinal mucosa is mediated by a type III secretion system (T3SS) [3], a molecular syringe that injects 21 effectors directly into the host cell cytosol where they hijack multiple cell signalling pathways, including those regulating cytoskeletal dynamics, inflammation, vesicle trafficking, and cell survival [4]. Multiple T3SS effectors trigger or antagonise host immune responses, including those mediated by nuclear factor kappa B (NF-κB) (e.g., translocated intimin receptor (Tir), NleF, NleC, NleD and NleE) [5–8], caspase-1 (NleA) [9], and caspase-4 (NleF) [10,11]. T3SS effector translocation is a highly regulated process which involves the transmembrane gatekeeper effector EspZ [12]; uncontrolled effector translocation is cytotoxic [12].

EPEC colonise intestinal epithelial cells (IECs) via a mechanism known as attaching and effacing (A/E) lesions, which are characterised by intimate bacterial attachment and effacing of the brush border microvilli [13]. Intimate EPEC attachment is mediated by avid interactions between the bacterial outer membrane adhesin intimin (encoded by the *eae* gene) and the effector Tir [14,15]. Binding to intimin leads to Tir clustering, phosphorylation of Tir tyrosine residue Y474 by host non-receptor tyrosine kinases [16,17], and recruitment of the host adaptor protein Nck, which, in turn, recruits N-WASP that activates Arp2/3 [18–20]. In addition, Tir Y454 forms a complex with phosphatidylinositol 3-kinase (PI3K) [21] and binds the host adaptors IRTKS/IRSp53, scaffold proteins that regulate actin organisation [22,23]. While the signalling downstream of Y474 cascade leads to robust actin polymerisation and formation of actin-rich pedestal-like structures at the site of bacterial attachment [18–20], pathway downstream of Y454 Triggers weak actin polymerisation activity [17,24]. Mutating both tyrosine residues to alanine or phenylalanine abolishes Tir phosphorylation and recruitment of host proteins involved in actin polymerisation [17,25].

Recently, in order to determine whether intimin–Tir interaction is necessary and sufficient for colonisation of the gut mucosa, we systematically deleted effector genes from the prototype EPEC strain E2348/69, generating EPEC-0 missing all the effector genes, EPEC-1 expressing only Tir and EPEC-2 that expresses only Tir and EspZ [26]. EPEC-0, 1, and 2 express intimin and a functional T3SS, while only EPEC-1 and EPEC-2 can trigger actin polymerisation in infected cells [26].

We have recently shown that intimin-induced Tir clustering, phosphorylation, and actin polymerisation trigger inflammasome-mediated pyroptotic cell death in EPEC-infected macrophages [25]. The inflammasome is a multi-protein complex which canonically involves caspase-1, the adaptor protein apoptosis-associated speck-like protein containing a CARD (ASC) and the nucleotide-binding oligomerisation domain (NOD) leucine-rich repeat proteins (NLRs), which act as sensors for pathogen/damage-associated molecular patterns (PAMPs/DAMPs) such as extracellular ATP, reactive oxygen species (ROS), bacterial toxins, and secretion system/flagella subunits [27–29]. The caspase-1 inflammasome cleaves and activates gasdermin D (GSDMD), a pore-forming protein that executes pyroptotic cell death [30,31] and secretion of interleukin (IL)-1β and IL-18 [25]. The noncanonical inflammasome comprises caspase-4, which is activated by direct binding to bacterial lipopolysaccharide (LPS) [30–32], known to enter the cytosol during bacterial invasion [33], via outer membrane vesicles (OMVs) [34,35] or via specific cellular receptors [36]. Recent studies have shown that the receptor-interacting serine/threonine-protein kinase 1 (RIPK1)/caspase-8 axis plays an important role in regulating apoptotic, necroptotic, and pyroptotic cell death pathways in IECs [37].

Our current understanding on the mechanisms of EPEC-induced cell death is mainly based on studies performed in macrophages. Nonetheless, pyroptosis can also be triggered in non-phagocytic IECs during bacterial infection [38]. Deep proteomic analysis of a panel of 50 intestinal cancer cell lines shows that caspase-4 and GSDMD are constitutively expressed, while NLR family pyrin domain containing 3 (NLRP3), gasdermin E (GSDME), and caspase-5 are undetectable under standard growth conditions [39]. The expression level of some of these pyroptotic proteins may be elevated via NF-κB or Janus kinase/signal transducers and activators of transcription (JAK-STAT) signalling pathways during a process called priming, via pretreatment with LPS or interferon gamma (IFNγ), respectively [28]. Although priming is most extensively used for phagocytic cells, IFNγ induces expression of caspase-1 and caspase-5 in the colonic epithelial cell line HT-29 [40]. Moreover, EPEC infection of the colorectal cancer cell line Caco-2 triggers caspase-4–dependent release of IL-18 [41], suggesting that EPEC may also induce pyroptosis in IECs. The aim of this study was to investigate whether EPEC causes pyroptosis in an IEC line model and to decipher its molecular mechanism.

## Results

### The interaction of EPEC with the colorectal epithelial cell line SNU-C5

**Fig 1:**
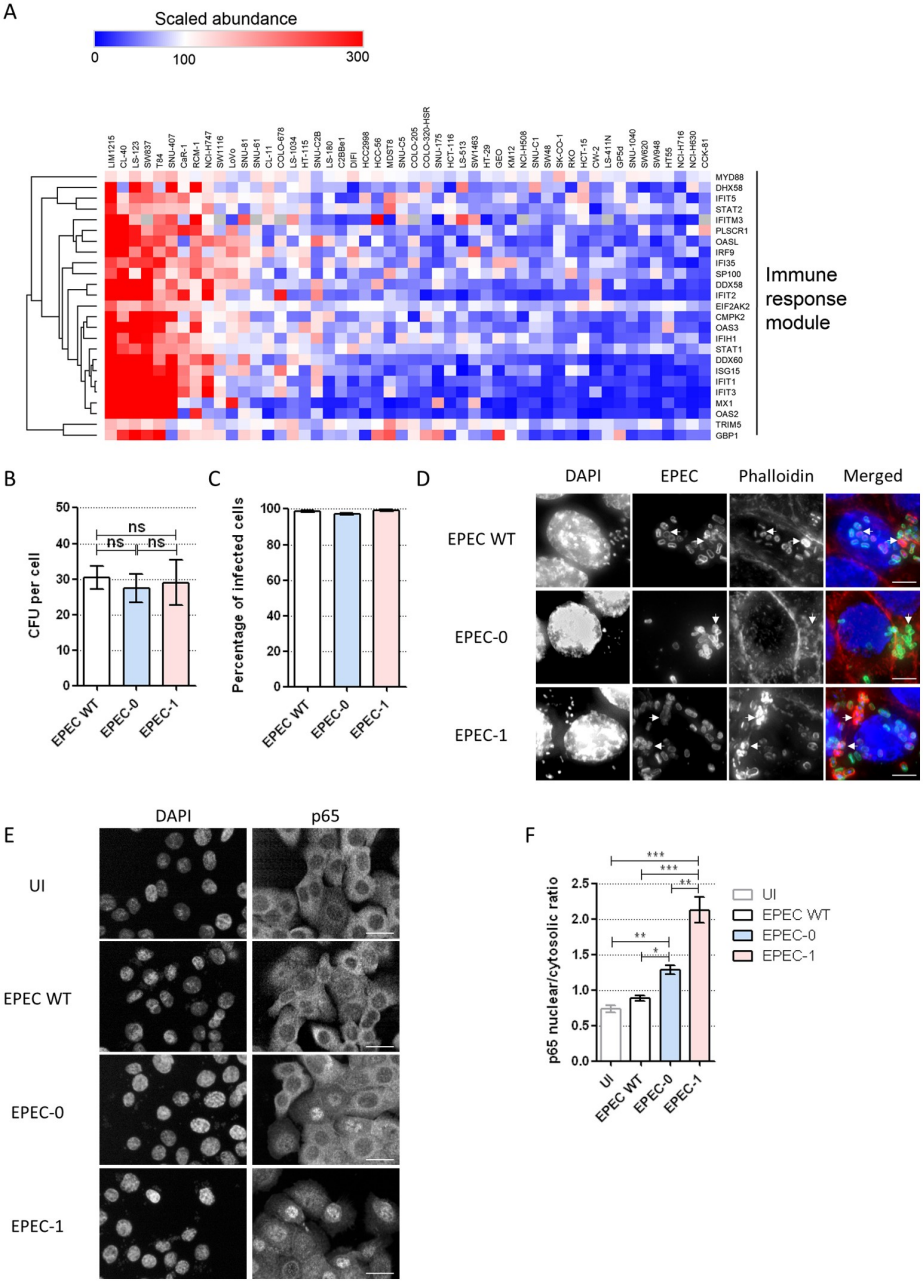
EPEC induces Tir-dependent actin polymerisation and NF-κB activation in SNU-C5 cells. (A) Ranking of 50 colorectal cell lines based on the scaled abundance of proteins in the immune response module according to WGCNA [39]. (B) Adhesion of EPEC WT, EPEC-0, and EPEC-1 to SNU-C5. Means ± SEM from *n* = 3 independent biological repeats. (C, D) Immunofluorescence labelling of SNU-C5 cells infected with EPEC WT, EPEC-0 and EPEC-1 or 4 h. (C) Percentage of infected cells. (D) Immunofluorescent images. DAPI: blue; EPEC: green; Phalloidin: red. Representative images from *n* = 3 independent biological repeats. Scale bar: 5 μm. Example bacteria are marked with white arrows. (E) Immunofluorescence staining of p65 in UI and EPEC-infected cells, analysed by high content imaging. Representative images from *n* = 3 independent biological repeats. Scale bar: 20 μm. (F) Nuclear/cytosolic ratio of the NF-κB p65 immunofluorescence intensity calculated by the Columbus-2 software. Statistical significance was determined using 1-way ANOVA with Tukey posttest. ns, nonsignificant; * *p* ≤ 0.05; ** *p* ≤ 0.01; *** *p* ≤ 0.001. The underlying data for this figure can be found in S1 Data. ANOVA, analysis of variance; DAPI, 4′,6-diamidino-2-phenylindole; EPEC, enteropathogenic *Escherichia coli*; NF-κB, nuclear factor kappa B; SEM, standard error of the mean; Tir, translocated intimin receptor; UI, uninfected; WGCNA, weighted correlation network analysis; WT, wild-type.

We aimed to investigate whether EPEC could trigger cell death in IECs and the potential role of IFNγ in this process. Most studies of EPEC infection have used either HeLa or Caco-2 cell lines; however, the latter contains several mutations in genes involved in IFNγ signalling pathway, including interferon gamma receptor 2 (IFNGR2) and Janus kinase 1 (JAK1). For this reason, we first sought to identify a suitable intestinal cell model for this study. Using publicly available proteomics data for a panel of 50 colorectal cancer cell lines [39], we analysed the abundance levels of proteins mapping in a characteristic immune response module, selected based on a weighted correlation network analysis (WGCNA) [42]. We ranked all 50 cell lines by the median scaled abundance of the immune response module [39] (Fig 1A) and selected the SNU-C5 cell line as a representative of moderate abundance of the proteins in this module. In addition, SNU-C5 cell line has no mutations in the genes involved in IFNγ signalling and noncanonical inflammasome: IFNGR1, IFNGR2, JAK1, JAK2, signal transducer and activator of transcription 1 (STAT1), interferon regulatory factor 1 (IRF1), IRF2, caspase-4 (CASP4), and GSDMD, according to the Colorectal Cancer Atlas [43]. We did not consider the variations of the canonical inflammasome components due to the undetectable protein expression levels of all known pyroptosis-related NLRs [39]. Additionally, the increased expression of guanylate-binding protein 2 (GBP2), an interferon-induced gene, in SNU-C5 primed with IFNγ for 24 h was validated by real-time quantitative PCR (qRT-PCR) (S1A Fig), confirming that SNU-C5 cells respond to IFNγ.

To further evaluate the cell model, we examined whether SNU-C5 cells are susceptible to EPEC infection. We used Tir-induced actin polymerisation and NF-κB activation as indicators of successful infection. SNU-C5 cells were infected with EPEC wild-type (WT), EPEC-1 (Tir only), and EPEC-0 (effectorless). While all 3 strains adhered to >98% of SNU-C5 cells (Fig 1B and 1C), robust actin polymerisation was detected underneath EPEC-WT and EPEC-1, but not EPEC-0 (Fig 1D). Immunofluorescence staining of NF-κB p65 subunit showed that EPEC-WT did not trigger NF-κB activation up to 4 h postinfection (Fig 1E and 1F), consistent with the notion that it expresses multiple effectors that inhibit the NF-κB pathway [6–8]. In contrast, EPEC-0, lacking these effectors, provoked modest NF-κB activation, which is likely due to the activity of the T3SS apparatus [44], while infection with EPEC-1 triggered significant NF-κB activation above EPEC-0 (Fig 1E and 1F). These results suggest that in the absence of the anti-inflammatory effectors and the gatekeeper EspZ [12], high-level Tir translocation in EPEC-1 triggers strong pro-inflammatory responses. Together, these data indicate that SNU-C5 responds as expected to EPEC infection and thus provides a robust model to study EPEC infection of IECs.

### EPEC-induced lytic cell death in SNU-C5 is enhanced by IFNγ priming

**Fig 2:**
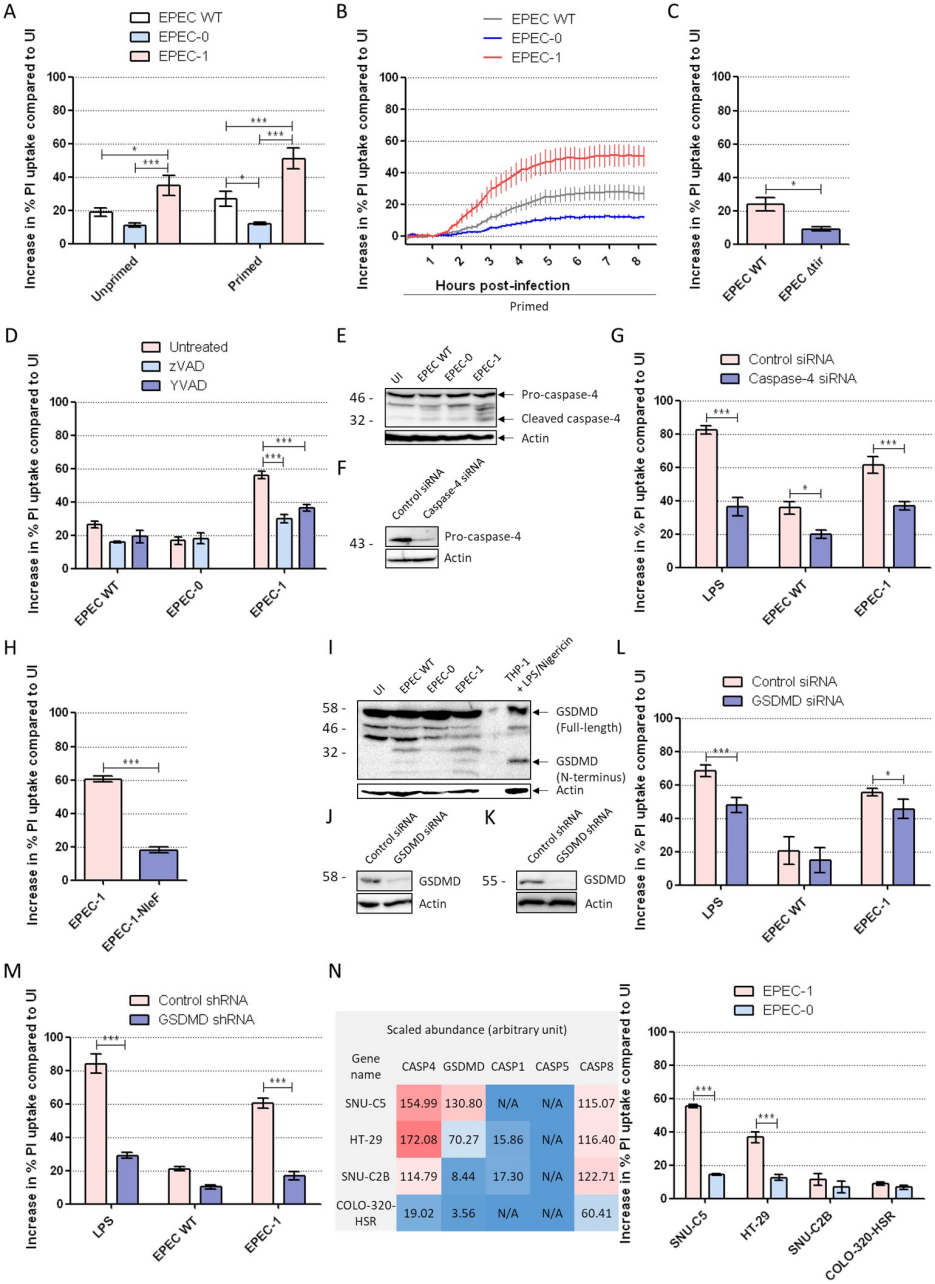
IFNγ enhances Tir- and caspase-4-dependent SNU-C5 cell death. (A) PI uptake into SNU-C5 cells infected with EPEC WT, EPEC-0, and EPEC-1 for 8 h. Cells were either unprimed or primed with 10 ng/ml of IFNγ. PI uptake results were normalised to uninfected cells. Means ± SEM from *n* = 5 independent biological repeats. (B) Time-course PI uptake into primed SNU-C5 cells throughout the 8 h of infection period. Measurements were taken every 10 min. Means ± SEM from *n* = 5 independent biological repeats are shown. (C) PI uptake into primed SNU-C5 cells infected with EPEC WT and Δ*tir*. Means ± SEM from *n* = 3 independent biological repeats. (D) PI uptake into primed SNU-C5 cells treated with zVAD and/or YVAD 30 min before infection with EPEC WT and EPEC-1. Means ± SEM from *n* = 3 (EPEC WT & EPEC-0) and 7 (EPEC-1) independent biological repeats. (E) Caspase-4 western blot of lysates and supernatants from primed cells infected by EPEC WT, EPEC-0, and EPEC-1. Representative blot from *n* = 3 independent biological repeats. (F) Caspase-4 western blot of lysates from primed cells transfected with caspase-4 siRNA for 2 days. Representative blot from *n* = 3 independent biological repeats are shown. (G) Control and caspase-4 siRNA transfection into primed SNU-C5 cells 2 days before infection. Shown is PI uptake following infection with EPEC WT and EPEC-1 or transfection with LPS. Means ± SEM from *n* = 4 (LPS & EPEC WT) and 7 (EPEC-1) independent biological repeats. (H) PI uptake into primed SNU-C5 cells infected with EPEC-1 and EPEC-1-NleF. Means ± SEM from *n* = 3 independent biological repeats. (I) GSDMD western blot of lysates from primed cells infected by EPEC WT, EPEC-0 and EPEC-1. Lysate of THP-1 cells after LPS/Nigericin treatment were used as positive control for GSDMD activation. Representative blot from *n* = 3 independent biological repeats. (J) GSDMD western blot of lysates from primed cells transfected with GSDMD siRNA for 3 days. Representative blot from *n* = 3 independent biological repeats are shown. (K) GSDMD western blot of lysates from primed GSDMD stable knockdown cells. Representative blot from *n* = 3 independent biological repeats are shown. (L) Control and GSDMD siRNA transfection into primed SNU-C5 cells 3 days before infection. Shown is PI uptake following infection with EPEC WT and EPEC-1 or transfection with LPS. Means ± SEM from *n* = 4 independent biological repeats. (M) Control and GSDMD stable knockdown cells were primed and transfected by LPS, infected by EPEC WT or EPEC-1. Means ± SEM from *n* = 3 independent biological repeats. (N) Left panel: the scaled endogenous abundance of caspase-4, GSDMD, caspase-1, 5 and 8 in the 4 cell lines [39]. N/A: undetectable. Right panel: PI uptake into primed SNU-C5, HT-29, SNU-C2B, and COLO-320-HSR cells infected with EPEC-0 and EPEC-1. Means ± SEM from *n* = 3 independent biological repeats. Statistical significance was determined using 2-way ANOVA with Bonferroni posttest (A, D, G, L, M, N) or 2-tailed *t* test (C, H). * *p* ≤ 0.05; ** *p* ≤ 0.01; *** *p* ≤ 0.001. The underlying data for this figure can be found in S1 Data. ANOVA, analysis of variance; EPEC, enteropathogenic *Escherichia coli*; GSDMD, gasdermin D; IFNγ, interferon gamma; LPS, lipopolysaccharide; PI, propidium iodide; SEM, standard error of the mean; siRNA, small interfering RNA; Tir, translocated intimin receptor; WT, wild-type; YVAD, z-YVAD-fmk; zVAD, z-VAD-fmk.

We next investigated whether EPEC infection induces death in SNU-C5 cells in a manner dependent on its effectors, using propidium iodide (PI) uptake as a reporter for membrane damage and necrotic cell lysis. This revealed that EPEC-WT induced 19% increase in PI uptake after 8 h of infection compared with uninfected control cells, while EPEC-0 increased PI uptake by 11% (Fig 2A, S1B Fig). Notably, EPEC-1 induced 35% increase in PI uptake, significantly higher than that of EPEC-WT and EPEC-0 (Fig 2A).

In order to determine the effect of IFNγ priming on EPEC-induced cell death, we pretreated the SNU-C5 monolayer with IFNγ 24 h prior to infection. IFNγ priming alone did not induce an increase in PI uptake (S1C Fig). Infecting the primed cells with EPEC-0 for 8 h showed no increase in PI uptake (12%), compared with unprimed cells. In contrast, IFNγ priming increased PI uptake to 27% and 51% following infection with EPEC-WT and EPEC-1, respectively (Fig 2A and 2B). The time course of PI uptake during EPEC infection revealed that cell death first started to increase at around 1.5 h and plateaued at 6 to 8 h postinfection (Fig 2B). To confirm that Tir is responsible for enhanced cell death, a control infection with EPECΔ*tir* revealed markedly reduced cell death compared with EPEC WT in primed cells (Fig 2C). Collectively, these results show that, similar to macrophages [25], Tir triggers cell death in an epithelial cell model and that this activity is amplified by IFNγ. We therefore decided to investigate EPEC-induced cell death mechanisms in IFNγ-primed cells.

### Tir-induced cell death is dependent on caspase-4 and GSDMD

To test the involvement of caspases in Tir-induced cell death in IECs, we pretreated SNU-C5 cells with either a pan-caspase inhibitor z-VAD-fmk (zVAD) or the pyroptotic caspase-1/4/5 inhibitor z-YVAD-fmk (YVAD). Both zVAD and YVAD significantly reduced cell death caused by EPEC-1 at 8 h postinfection, indicating the involvement of pyroptosis, while the low level of Tir-independent cell death in EPEC-0 was unaffected by caspase inhibition (Fig 2D). Furthermore, western blot of infected cells showed no cleavage of the apoptosis marker poly [ADP-ribose] polymerase 1 (PARP1), ruling out apoptosis in Tir-dependent cell death (S2A Fig). In contrast to macrophage [25], Tir-dependent cell death in SNU-C5 was not inhibited by the NLRP3 inhibitor MCC950 (S2B and S2C Fig). As *Yersinia*, a related Gram-negative pathogen carrying T3SS, has recently been shown to trigger macrophage pyroptosis via RIPK1- and caspase-8-dependent cleavage of GSDMD [45, 46, 47], we infected SNU-C5 cells in the presence of the RIPK1 inhibitor Nec1 (S2D–S2F Fig) or with caspase-8 small interfering RNA (siRNA) silencing (S2G and S2H Fig), both of which revealed no inhibition of cell death.

The pan-caspase inhibition did not completely abolish Tir-dependent cell death, with *ca*. 15% of EPEC-1-infected cells sill exhibiting PI uptake compared with EPEC-0-infected cells (Fig 2D). As necroptosis can be activated during pyroptosis inhibition [48], we investigated if the residual cell death is due to necroptosis. For this, we used necrostatin-1 (Nec1) to inhibit RIPK1, necrosulfonamide (NSA) to inhibit MLKL and siRNA to silence RIPK3. While control experiments have shown that these treatments prevented necroptosis induced by staurosporine (STS) and zVAD co-stimulation [49], they did not significantly affect Tir-dependent cell death, either on their own or in combination with zVAD or caspase-4 silencing (S2D–S2K Fig). Taken together, these results implicate the pyroptotic caspases as the main contributor in EPEC-induced cell death, while the mechanism leading to the residual cell death remains elusive.

As neither caspase-1 nor caspase-5 is detected in SNU-C5 [39], we next investigated the involvement of caspase-4 which is detected in the proteome. Western blotting of cell lysates and supernatants collected at 8 h postinfection revealed cleavage of the approximately 45 kDa pro-caspase-4 into the active form approximately 30 kDa caspase-4 large subunit in EPEC-1-infected cells (Fig 2E). To verify its functional role, we silenced caspase-4 by siRNA, which reduced cell death when LPS transfection was used as a positive control (Fig 2F and 2G). Upon infection with either EPEC WT or EPEC-1, cell death was suppressed by caspase-4 siRNA (Fig 2G), suggesting that Tir-induced cytotoxicity is mediated by caspase-4-driven pyroptosis. Indeed, ectopic expression of NleF, which inhibits caspase-4 [10,11], in EPEC-1 resulted in strong inhibition of Tir-induced cell death (Fig 2H). Moreover, EPEC-1-infected cells exhibited higher levels of cleaved N-terminal GSDMD fragment that is responsible for the pyroptotic pores (Fig 2I). Silencing GSDMD in SNU-C5 by siRNA only mildly reduced EPEC-1-induced cell death (Fig 2J and 2L). Consequently, we constructed GSDMD stable knockdown SNU-C5 cell line using the microRNA30E short-hairpin RNA (shRNA) strategy [25,50]. GSDMD shRNA strongly reduced cell death by the positive control LPS transfection as well as both EPEC WT and EPEC-1, confirming that GSDMD is required for Tir-dependent cell death (Fig 2K and 2M).

To further verify the dependence of cell death on GSDMD, we selected 3 additional IEC lines, HT-29, SNU-C2B, and COLO-320-HSR, in which the abundance of GSDMD is 1.9, 15.5, and 36.7 times lower compared with SNU-C5, while the abundance ratios for caspase-4 of these cell lines over SNU-C5 are 1.1, 0.7, and 0.1, respectively (Fig 2N, left panel). These cell lines display no mutations in either caspase-4 or GSDMD genes. Caspase-5 was undetectable, and caspase-8 was expressed in all cell lines, while caspase-1 was only detected at a low level in HT-29 and SNU-C2B cells (Fig 2N, left panel). Adhesion of EPEC WT, EPEC-0, and EPEC-1 and Tir translocation was confirmed in all cell lines (S3 Fig). Infections of IFNγ-primed SNU-C5 and HT-29 with EPEC-1 resulted in higher cell death compared with those with EPEC-0 (Fig 2N, right panel). In contrast, EPEC-0 caused similar lower level of cell death upon infection of COLO-320-HSR and SNU-C2B (Fig 2N, right panel). These results reveal a direct and specific correlation between the endogenous level of GSDMD and Tir-dependent cell death.

### Tir clustering triggers epithelial cell death

**Fig 3:**
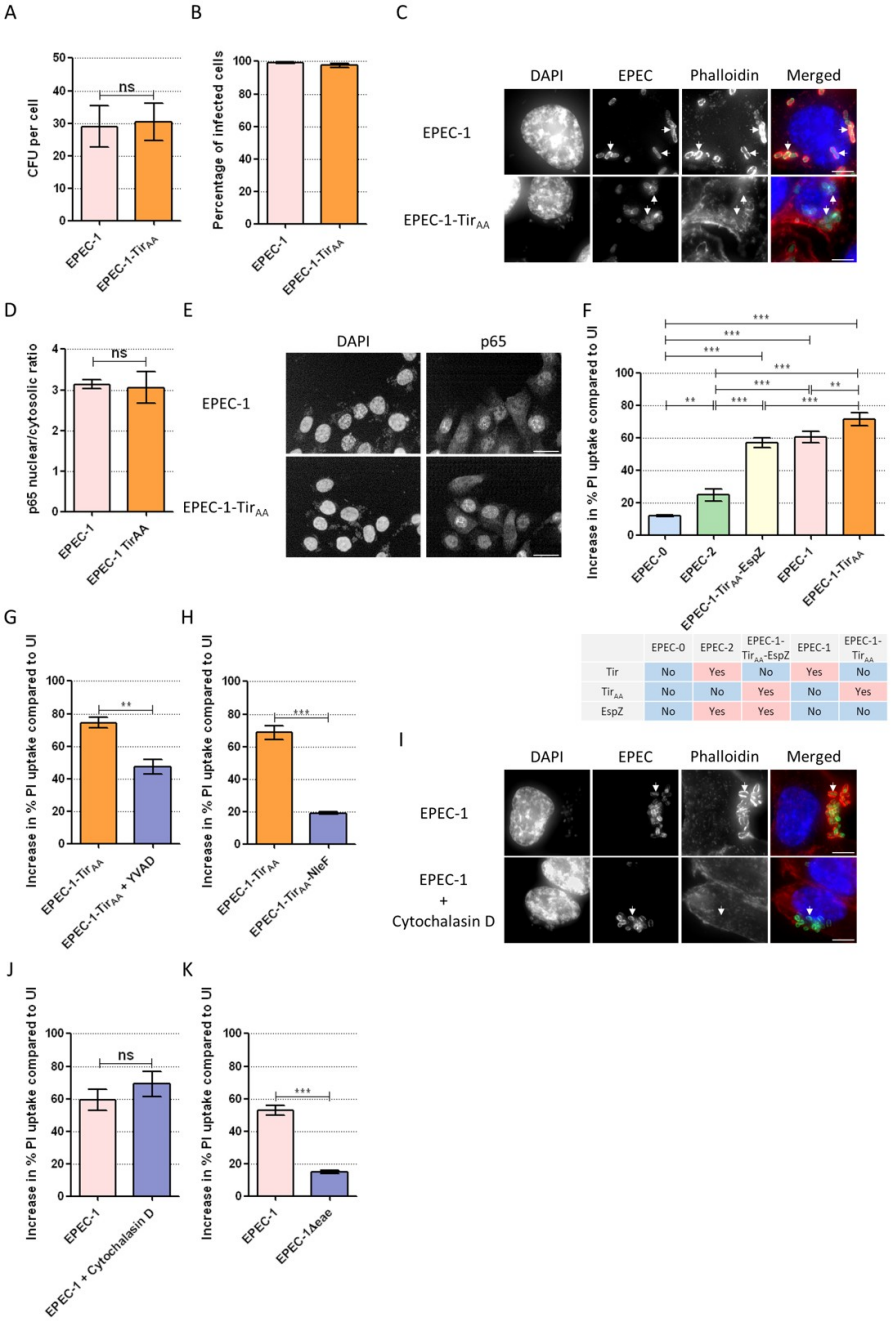
Clustering by intimin is essential for Tir-induced cell death. (A) Infection rates of EPEC-1 and EPEC-1-Tir_AA_ on SNU-C5 cells. Means ± SEM from *n* = 3 independent biological repeats. (B, C) Immunofluorescence labelling of SNU-C5 cells infected with EPEC-1 and EPEC-1-Tir_AA_ for 2 h. (B) Percentage of infected cells. (C) DAPI: blue; EPEC: green; Phalloidin: red. Representative images from *n* = 3 independent biological repeats. Scale bar: 5 μm. Example bacteria are marked with white arrows. (D, E) Nuclear/cytosolic ratio of the NF-κB p65 immunofluorescence intensity calculated by the Columbus-2 software (D) and representative images (E) from *n* = 3 independent biological repeats of unprimed SNU-C5 cells infected with EPEC-1 and EPEC-1-Tir_AA_. Scale bar: 20 μm. (F–H) PI uptake into primed SNU-C5 cells infected with EPEC-0, EPEC-2, EPEC-1-Tir_AA_-EspZ (F), EPEC-1 (F, I), EPEC-1-Tir_AA_ (F–H), and EPEC-1-Tir_AA_-NleF (H). The effector compositions in EPEC-0, EPEC-2, EPEC-1-Tir_AA_-EspZ, EPEC-1, and EPEC-1-Tir_AA_ are listed (F). Primed SNU-C5 cells were treated by YVAD and infected with EPEC-1-Tir_AA_ and used for PI uptake assays. Means ± SEM from *n* = 5 (F) and 3 (G-H) independent biological repeats. (I) Immunofluorescence labelling of SNU-C5 cells infected with EPEC-1 in the presence of absence of cytochalasin D treatment. DAPI: blue; EPEC: green; Phalloidin: red. Representative images from *n* = 4 independent biological repeats. Scale bar: 5 μm. Example bacteria are marked with white arrows. (J) PI uptake into EPEC-1-infected SNU-C5 cells in the presence of absence of cytochalasin D. Means ± SEM from *n* = 3 independent biological repeats. (K) PI uptake into SNU-C5 cells infected by EPEC-1 or EPEC-1 Δ*eae*. Means ± SEM from *n* = 3 independent biological repeats. Statistical significance was determined using 2-tailed *t* test (A, D, G, H, J, K) or 1-way ANOVA with Tukey posttest (F). * *p* ≤ 0.05; ** *p* ≤ 0.01; *** *p* ≤ 0.001. The underlying data for this figure can be found in S1 Data. ANOVA, analysis of variance; DAPI, 4′,6-diamidino-2-phenylindole; EPEC, enteropathogenic *Escherichia coli*; NF-κB, nuclear factor kappa B; PI, propidium iodide; SEM, standard error of the mean; Tir, translocated intimin receptor; YVAD, z-YVAD-fmk.

Binding of intimin to Tir leads to its clustering and activation of a robust actin polymerisation cascade, which is essential for triggering cell death in human macrophages [25]. In order to determine whether Tir-induced actin polymerisation is also necessary for epithelial cell death, we generated a Tir_Y454A/Y474A_ mutant in EPEC-1 (EPEC-1-Tir_AA_), which should not induce actin polymerisation. As controls, we confirmed that EPEC-1-Tir_AA_ adhered to SNU-C5 cells similar to EPEC-1 (Fig 3A and 3B), did not induce detectable actin polymerisation (Fig 3C), and triggered p65 nuclear translocation at a level comparable with EPEC-1 (Fig 3D and 3E). Importantly, contrary to the observation in human macrophages [25], EPEC-1-Tir_AA_ still induced pyroptosis following infection of SNU-C5 cells (Fig 3F). Both YVAD and ectopic expression of NleF markedly reduced EPEC-Tir_AA_-induced cell death, suggesting that like WT Tir, Tir_AA_ activates caspase-4 in this model (Fig 3G and 3H). To further confirm that actin polymerisation is dispensable for cell death, we pretreated SNU-C5 cells with the actin polymerisation inhibitor cytochalasin D, which prevented actin pedestal formation but not cell death (Fig 3I and 3J). To assess the role of Tir clustering, we used a deletion mutant lacking intimin from EPEC-1 (EPEC-1Δ*eae*) [26]. The infection of SNU-C5 cells with EPEC-1Δ*eae* resulted in significant reduction of cell death (Fig 3K). This implied that while intimin-induced Tir clustering is essential, the actin polymerisation function of Tir is dispensable for induction of cell death in IECs.

EspZ has been shown to limit the level of Tir translocation [12]. In order to determine if the magnitude of cell death is impacted by the level of Tir translocation, we measured cell death induced by EPEC-2 (EPEC-1 expressing EspZ) and EPEC-1-Tir_AA_ ectopically expressing EspZ (EPEC-1-Tir_AA_-EspZ). EPEC-2 and EPEC-1-Tir_AA_-EspZ induced significantly less cell death compared with EPEC-1 and EPEC-1-Tir_AA_, respectively (Fig 3F). Notably, the relatively high level of cell death in EPEC-1-Tir_AA_- and EPEC-1-Tir_AA_-EspZ-infected cells compared with EPEC-1- and EPEC-2-infected cells, respectively (Fig 3F), as well as the mild increase in cell death caused by cytochalasin D, suggests that the loss of actin polymerisation ability enhances cell death. Based on these data, we conclude that the dosage of translocated Tir impacts on cell death. Moreover, it shows a strain-dependent gradient of EPEC-induced cell death in both primed and unprimed cells: EPEC-0 < EPEC-2 < EPEC-1-Tir_AA_-EspZ < EPEC-1 < EPEC-1-Tir_AA_ (Fig 3F, S3C Fig).

### Tir affects the abundance of Ca^2+^ transport and response proteins

**Fig 4:**
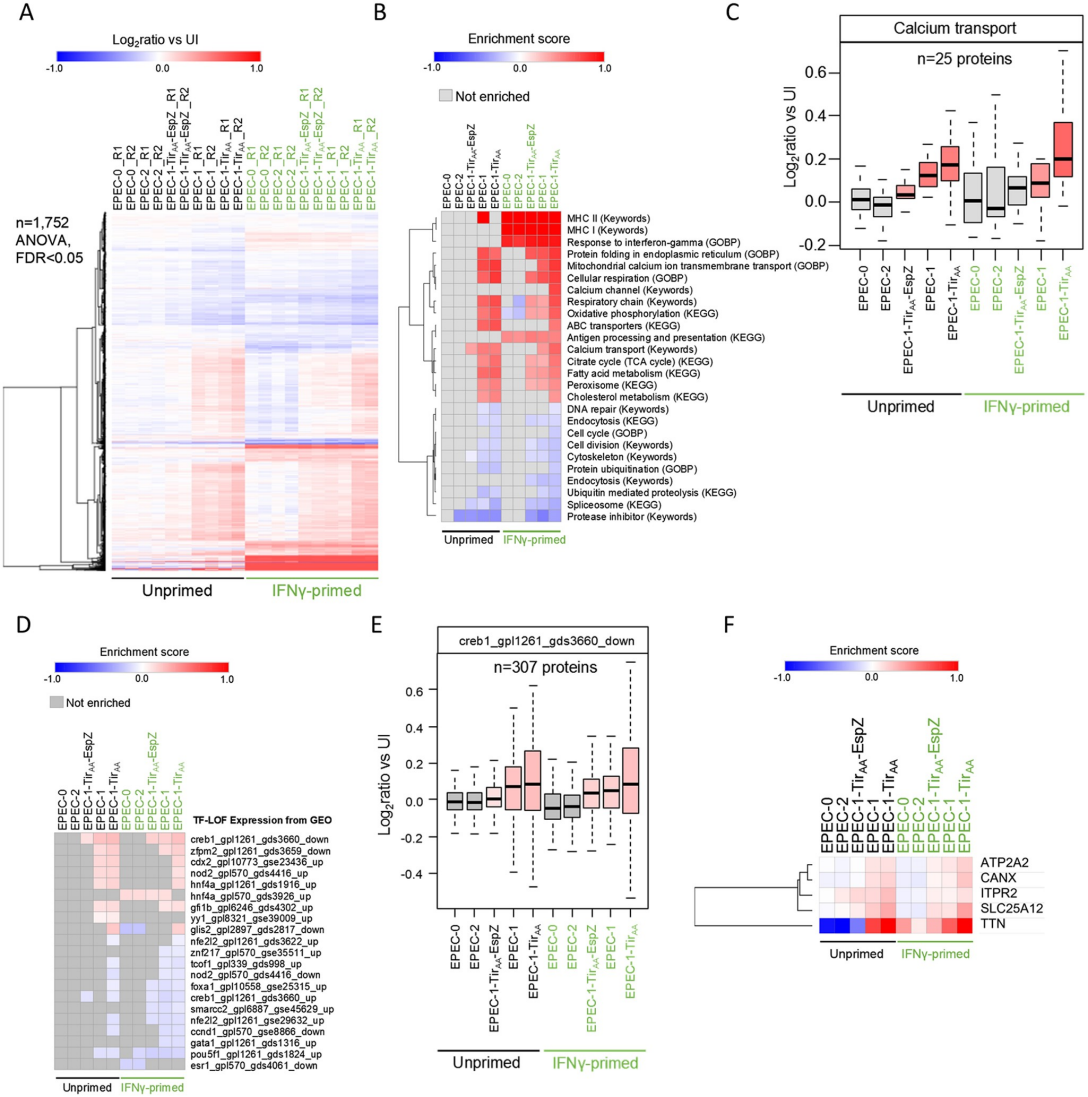
Proteomic analysis of SNU-C5 infected with EPEC variants inducing variable levels of cell death. (A) Hierarchical clustering (Euclidean distance) of differentially regulated proteins (ANOVA, FDR < 0.05) between EPEC-0, EPEC-2, EPEC-1-Tir_AA_-EspZ, EPEC-1, and EPEC-1-Tir_AA_-infected cells with and without INFNγ priming. Data represent log_2_ ratios versus the uninfected sample and 2 independent biological repeats were performed per infection. Individual repeats are labelled in the heatmap (R1 and R2). (B) Representative enriched pathways shown as a heatmap of the enrichment scores (Benjamini–Hochberg FDR < 0.05). (C) Box plot of changes in Ca^2+^ transporter proteins selected using Keywords across the different samples. The boxes are color-coded according to the enrichment scores shown in panel B. (D) Transcription factor enrichment was performed using the “TF-LOF Expression from GEO” library. (E) Box plot of protein changes of a gene set with known reduced expression upon CREB1 perturbation (therefore positively regulated by CREB1). These proteins tend to be up-regulated in our data set, suggesting the activation of CREB1. The boxes are color-coded according to the enrichment scores shown in panel D. (F) Hierarchical clustering of proteins selected by creb1_gpl1261_gds3660_down (TF-LOF Expression from GEO) and Calcium (Keywords). The underlying data for this figure can be found in S1 Data. ANOVA, analysis of variance; CREB1, cAMP responsive element binding protein 1; EPEC, enteropathogenic *Escherichia coli*; FDR, false discovery rate; IFNγ, interferon gamma; GEO, Gene Expression Omnibus; TF-LOF, transcription factor loss of function; Tir, translocated intimin receptor.

In order to gain mechanistic insights of Tir-induced caspase-4 activation, we performed proteomics analysis of SNU-C5 cells following infection of unprimed and IFNγ-primed cells with EPEC-0, EPEC-2, EPEC-1-Tir_AA_-EspZ, EPEC-1, and EPEC-1-Tir_AA_, using isobaric labelling (S5A Fig). We selected 2 h postinfection for the analysis representing the time point where phenotypic signs of cell death are evident but with limited cell loss (Fig 2B, S1B Fig). We quantified a total of 8,888 human proteins, of which 1,752 were found differentially regulated between the different infections (analysis of variance [ANOVA], adjusted *P* < 0.05). Principal component analysis (PCA) using all proteins showed a strong separation by IFNγ priming as well as by the strain-dependent gradient of cell death, with the largest variation observed between EPEC-0/EPEC-2 and EPEC-1-Tir_AA_ for both primed and unprimed cells (S5B Fig). Hierarchical clustering revealed groups of differentially regulated proteins either positively or negatively correlating with the cell death gradient as well as clusters of proteins regulated by IFNγ-priming in an infection-independent manner (Fig 4A). Enrichment analysis identified a diverse range of signalling pathways and biological processes with up- or down-regulation trends across infections (Fig 4B). As expected, proteins involved in antigen presentation and response to IFNγ were highly up-regulated in all primed samples (Fig 4B). A heatmap of the 106 differentially regulated proteins upon IFNγ priming, including caspase-4 and GSDMD, is shown in S5 Fig. Several basic cellular processes including, proteolysis, spliceosome, cell cycle, and cytoskeleton proteins showed a decreased abundance that is correlated with Tir-induced death profile (Fig 4B). Strikingly, Ca^2+^ transport, protein folding in endoplasmic reticulum (ER), and mitochondrial metabolism were among the most up-regulated pathways by EPEC-1 and EPEC-1-Tir_AA_, and to a lesser extent by EPEC-1-Tir_AA_-EspZ, compared with EPEC-2 and EPEC-0 (Fig 4B). In particular, Ca^2+^ transport proteins showed a clear trend of up-regulation correlating with the cell death gradient (Fig 4C).

To investigate whether any of the observed proteomic changes could be explained by early activation or inhibition of upstream factors, we performed enrichment analysis using expression gene sets from transcription factor perturbations from the Gene Expression Omnibus (GEO) database [51]. This analysis identified cAMP responsive element binding protein (CREB1) as the most significantly enriched transcription factor with enrichment scores positively correlating with the cell death gradient (Fig 4D and 4E). CREB1 is activated by Ca^2+^ influx and subsequently regulates the downstream expression of Ca^2+^ transport proteins as a feedback response [52,53]. Proteins related to Ca^2+^ signalling, including the ER Ca^2+^ transporters ATP2A2 (1 of the sarco/endoplasmic reticulum Ca^2+^ ATPases (SERCA); the main ER Ca^2+^ importer) and inositol 1,4,5-trisphosphate receptor type 2 (ITPR2, ER Ca^2+^ release channel), Ca^2+^ response protein calnexin (CANX) which senses and regulates Ca^2+^ oscillations, and several other Ca^2+^-binding proteins, were also predicted downstream of CREB1 signalling and show changes in expression similar to the cell death gradient (Fig 4F).

In addition, ER and mitochondria are both major Ca^2+^ storage sites and modulated by Ca^2+^ signalling. Many enzymes involved in protein folding in ER and mitochondrial metabolism require Ca^2+^ binding and are regulated by Ca^2+^ homeostasis [54,55]. Therefore, it is likely that the up-regulation of ER protein folding and mitochondrial metabolism proteins during EPEC infection resulted from enhanced Ca^2+^ signalling. Importantly, caspase-4 can be activated by increased intracellular Ca^2+^ level, for example, ER Ca^2+^ release mediated by transmembrane protein 173 (TMEM173) (also known as stimulator of interferon genes (STING)) [56] or the SERCA inhibitor thapsigargin [57]. Based on our current findings and published data, we hypothesised that a change in Ca^2+^ concentration in the cytosol modulates the viability of the infected cells.

### Ca^2+^ influx mediates Tir-induced cell death

**Fig 5:**
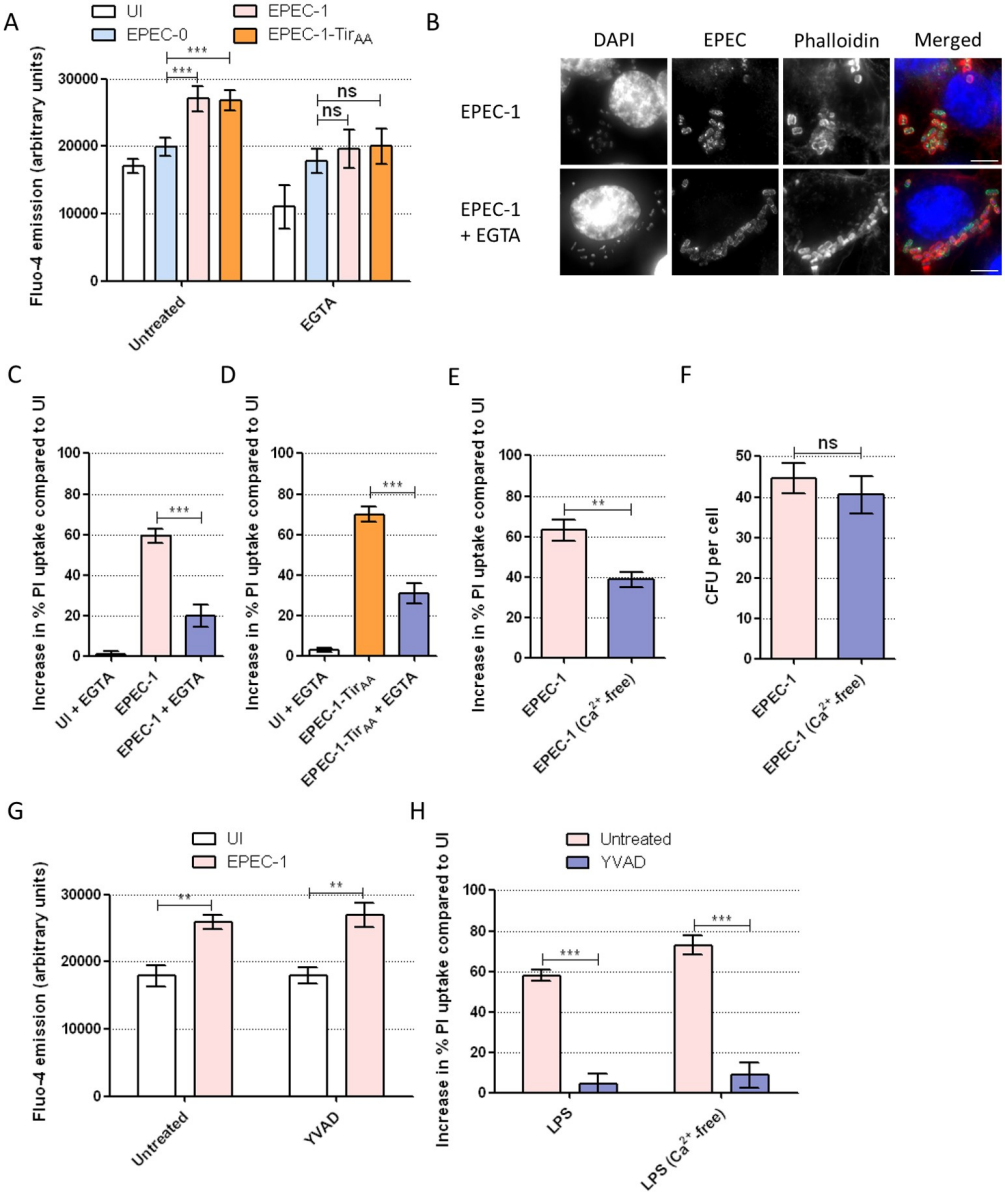
Tir-induced extracellular Ca^2+^ influx promotes cell death. (A) Fluo-4 assay of primed SNU-C5 cells infected with EPEC-0, EPEC-1 and EPEC-1-Tir_AA_ with or without 30 min pretreatment with EGTA. The Fluo-4 emission at 40 min postinfection was plotted. Means ± SEM from *n* = 3 independent biological repeats. (B) Immunofluorescence labelling of SNU-C5 cells infected with EPEC-1 for 2 h with and without EGTA pretreatment. DAPI: blue; EPEC: green; Phalloidin: red. Representative images from 3 independent biological repeats are shown. Error bar: 5 μm. (C, D) PI uptake into SNU-C5 cells infected with EPEC-1 (C) or EPEC-1-Tir_AA_ (D) with or without 30 min pretreatment with EGTA. Means ± SEM from *n* = 3 independent biological repeats. (E) PI uptake into SNU-C5 cells grown in complete DMEM or Ca^2+^-free DMEM infected with EPEC-1. Means ± SEM from *n* = 4 independent biological repeats. (F) Adhesion of EPEC-1 to SNU-C5 cells grown in complete or Ca^2+^-free DMEM. Means ± SEM from *n* = 3 independent biological repeats. (G) Fluo-4 of SNU-C5 cells infected with EPEC-1 with or without 30 min pretreatment with YVAD. The Fluo-4 emission at 40 min postinfection was plotted. Means ± SEM from *n* = 3 independent biological repeats. (H) PI uptake into SNU-C5 cells grown in complete or Ca^2+^-free DMEM transfected with LPS with or without 30 min pretreatment with YVAD. Means ± SEM from *n* = 3 independent biological repeats. Statistical significance was determined using 2-way ANOVA with Bonferroni posttest (A, G, H) or 2-tailed *t* test (C-F). * *p* ≤ 0.05; ** *p* ≤ 0.01; *** *p* ≤ 0.001. The underlying data for this figure can be found in S1 Data. ANOVA, analysis of variance; DAPI, 4′,6-diamidino-2-phenylindole; EGTA, ethylene glycol-bis(β-aminoethyl ether)-N,N,N′,N′-tetraacetic acid; EPEC, enteropathogenic *Escherichia coli*; PI, propidium iodide; SEM, standard error of the mean; Tir, translocated intimin receptor; YVAD, z-YVAD-fmk.

Based on the changes in the abundance of the plasma membrane Ca^2+^ influx transporters and the ER Ca^2+^ importer ATP2A2 along the cell death gradient (Fig 4), we hypothesised that EPEC infection leads to Ca^2+^ influx. To test this hypothesis, we used the Fluo-4 Ca^2+^ reporter to measure cytosolic-free Ca^2+^ levels. We found that 40 min postinfection with EPEC-1 and EPEC-1-Tir_AA_, SNU-C5 cells exhibit increased cytosolic Ca^2+^ levels compared with EPEC-0-infected and uninfected cells (Fig 5A, S6A Fig). In addition, Tir-dependent Ca^2+^ influx was detected in unprimed cells infected with either EPEC-1 or EPEC-1-Tir_AA_ at similar level to primed cells, suggesting IFNγ treatment has negligible effect on Ca^2+^ influx (S7B Fig).

Although mitochondrial proteins were up-regulated and ROS generated from respiration as a result of Ca^2+^ influx could lead to cell death [58], we found that the ROS scavenger and the respiration inhibitor oligomycin had no significant effect on Tir-induced cell death (S8 Fig).

In order to investigate the source of Ca^2+^ influx in cell death, we pretreated SNU-C5 cells with extracellular Ca^2+^ chelating agent EGTA. This completely diminished the increase in cytosolic Ca^2+^ and resulted in the attenuation of pyroptosis induced by both EPEC-1 and EPEC-1-Tir_AA_ (Fig 5C and 5D; S7A and S7B Fig), without affecting EPEC-1-mediated actin polymerisation (Fig 5B). Moreover, cell death induced by EPEC-1 was significantly lower when SNU-C5 cells were grown in Ca^2+^-free, compared with complete DMEM media (Fig 5E). Enumeration of CFU showed similar bacterial attachment in Ca^2+^-free medium (Fig 5F). While the GSDMD pore has been previously shown to induce Ca^2+^ influx [59], YVAD did not affect Tir-induced Ca^2+^ influx, suggesting that this event lies upstream of caspase-4 and GSDMD cleavage (Fig 5G, S7C Fig). Notably, cell death caused by LPS transfection was not reduced in Ca^2+^-free DMEM (Fig 5H), indicating that extracellular Ca^2+^ influx is not necessary for activation of caspase-4 by cytosolic LPS.

**Fig 6:**
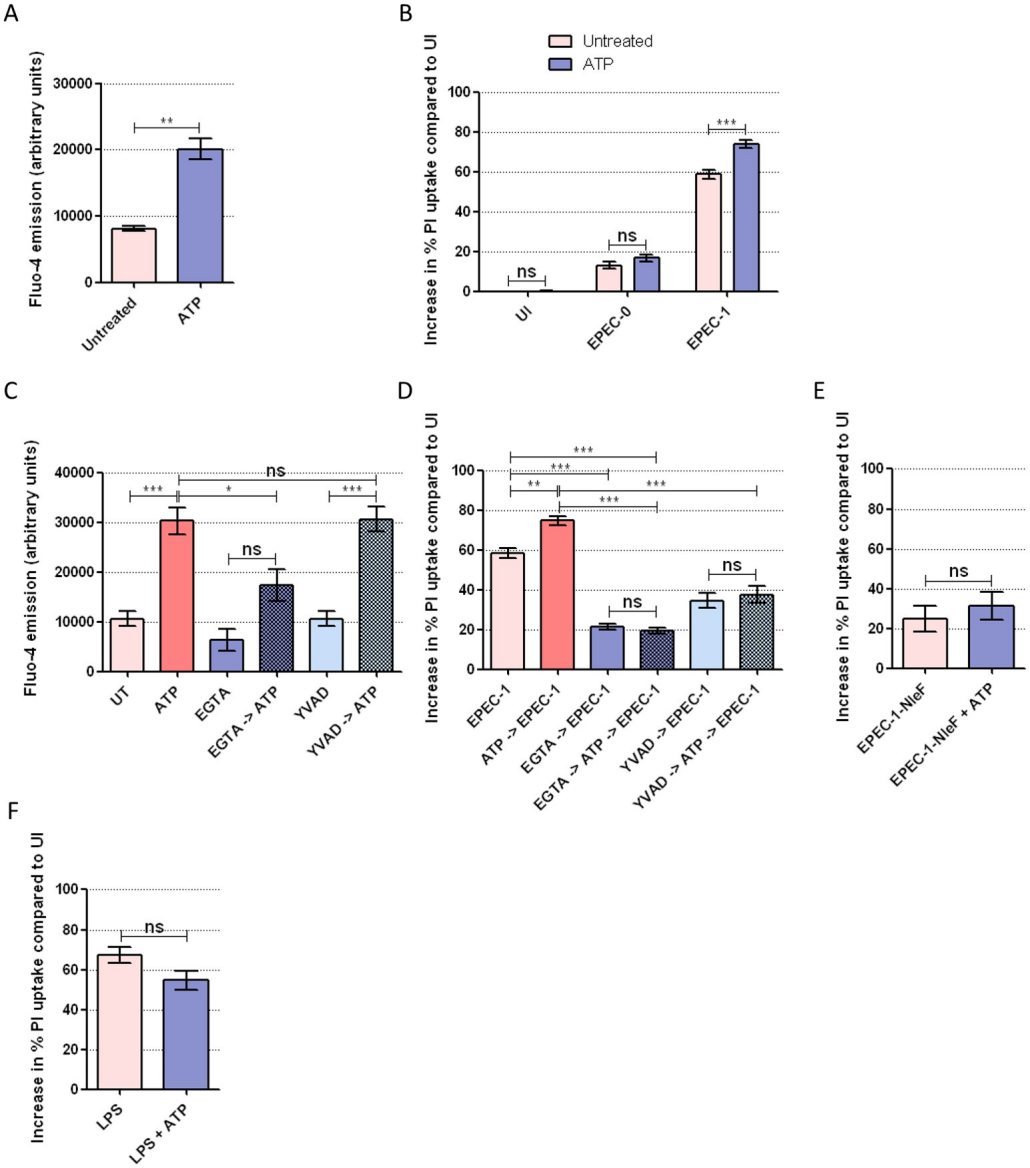
Pharmacologically induced Ca^2+^ influx promotes Tir-dependent cell death upstream of pyroptotic caspase activation. (A) Fluo-4 assay of SNU-C5 cells with or without 2 min pretreatment with ATP. Means ± SEM from *n* = 3 independent biological repeats. (B) PI uptake into SNU-C5 cells infected with EPEC-0 or EPEC-1, with or without 30 min pretreatment with ATP. Means ± SEM from *n* = 6 (UI and EPEC-1) and 3 (EPEC-0) independent biological repeats. (C, D) PI uptake into SNU-C5 cells infected with EPEC-1 with 30 min pretreatment with ATP, EGTA, or YVAD or 45 min pretreatment with EGTA or YVAD, followed by ATP for 30 min. The order of treatment in each sample is indicated by the arrow. Fluo-4 assay was performed on the uninfected cells with the same treatment at 2 min after ATP addition. Means ± SEM from *n* = 5 (EPEC-1 and EPEC-1 + ATP in D) and 3 (C, D) independent biological repeats. (E) PI uptake into SNU-C5 cells infected with EPEC-1 and EPEC-1-NleF with or without 30 min pretreatment with ATP. Means ± SEM from *n* = 3 independent biological repeats. (F) PI uptake into SNU-C5 cells transfected with LPS with or without pretreatment of ATP for 30 min. Means ± SEM from *n* = 4 independent biological repeats are shown. Statistical significance was determined using 2-way ANOVA with Bonferroni posttest (A), 2-tailed *t* test (B, E, F) or 1-way ANOVA with Tukey posttest (C, D). ns, nonsignificant; * *p* ≤ 0.05; ** *p* ≤ 0.01; *** *p* ≤ 0.001. The underlying data for this figure can be found in S1 Data. ANOVA, analysis of variance; ATP, adenosine triphosphate; EGTA, ethylene glycol-bis(β-aminoethyl ether)-N,N,N′,N′-tetraacetic acid; EPEC, enteropathogenic *Escherichia coli*; PI, propidium iodide; SEM, standard error of the mean; Tir, translocated intimin receptor; YVAD, z-YVAD-fmk.

To further substantiate the role of Ca^2+^ in cell death, we used ATP, an agonist of the Ca^2+^-permeable purinergic channels [60], to pharmacologically induce Ca^2+^ influx before EPEC infection. First, to confirm the effect of ATP on Ca^2+^ influx on SNU-C5 cells, we performed Fluo-4 assay on cells treated with 0.5 mM ATP, which revealed increased Ca^2+^ influx within 2 min after ATP treatment (Fig 6A). We then performed PI uptake assays on EPEC-0 and EPEC-1-infected cells pretreated with ATP for 30 min. ATP treatment significantly enhanced cell death by EPEC-1 but not EPEC-0, while ATP alone did not affect the viability of uninfected cells (Fig 6B). Therefore, although Ca^2+^ augments Tir-dependent cell death, it cannot substitute Tir. Furthermore, treating cells with EGTA before the addition of ATP reduced the rapid Ca^2+^ uptake and Tir-induced cell death (Fig 6C and 6D). To study whether ATP-induced Ca^2+^ influx enhances Tir-dependent cell death via caspase-4, we treated the cells with YVAD which by itself did not inhibit Ca^2+^ influx (Fig 6C). YVAD prevented ATP-dependent increase in Tir-induced cell death, suggesting that increased Ca^2+^ influx cannot bypass the need of caspases (Fig 6D). In support of this, we also found that ATP failed to promote Tir-induced cell death in the presence of NleF (Fig 6E). Importantly, ATP pretreatment did not elevate cell death caused by LPS transfection (Fig 6F). Hence, Ca^2+^ influx specifically promotes Tir-induced cell death.

### Tir induces Ca^2+^-dependent pyroptosis on a primary epithelial cell line

**Fig 7:**
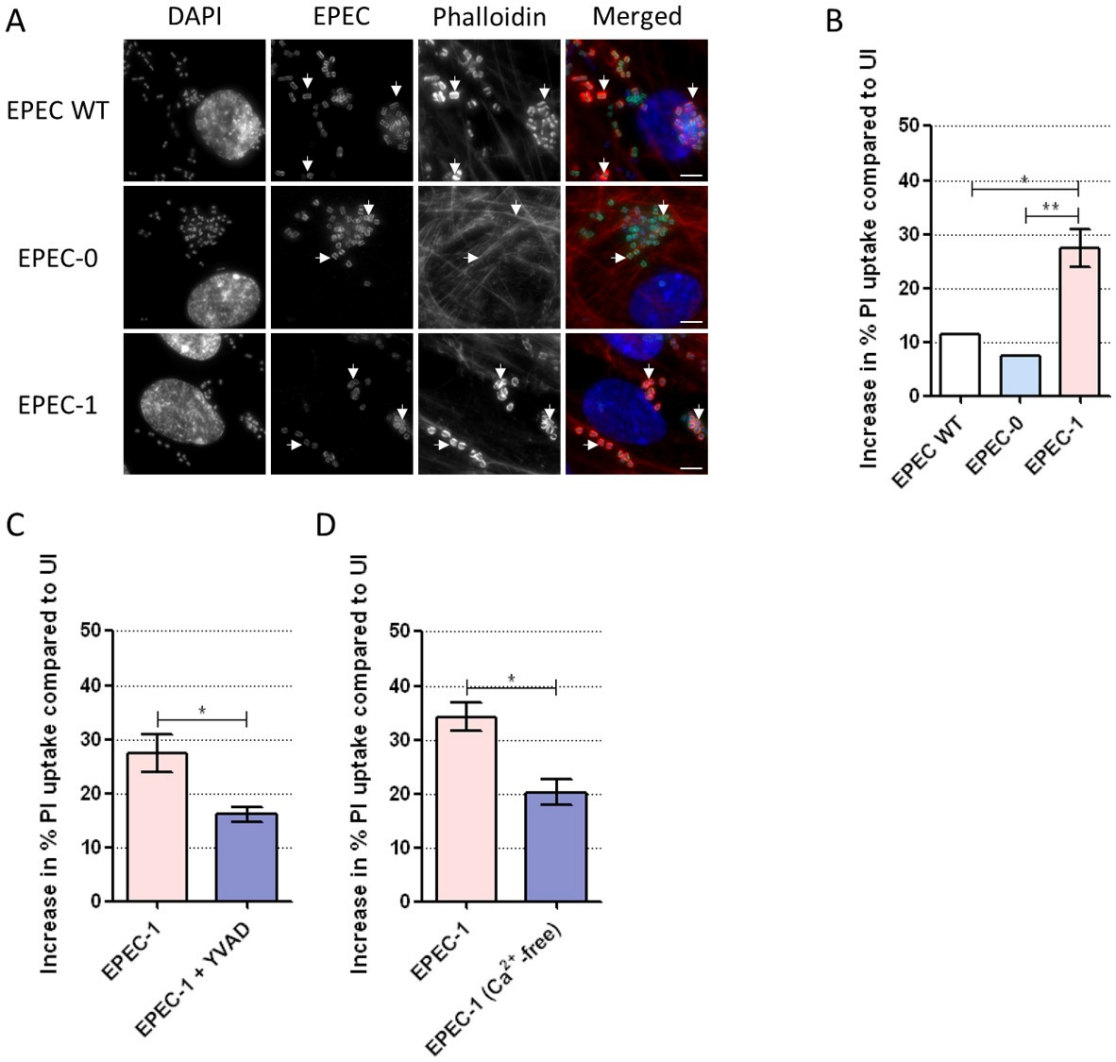
EPEC induces Tir- and Ca^2+^-dependent pyroptosis in RPE cells. (A) Immunofluorescence labelling of RPE cells infected with EPEC WT, EPEC-0 and EPEC-1 for 2 h. DAPI: blue; EPEC: green; Phalloidin: red. Representative images from *n* = 3 independent biological repeats. Scale bar: 5 μm. Example bacteria are marked with white arrows. (B) PI uptake into primed RPE cells infected with EPEC WT, EPEC-0, and EPEC-1 for 8 h. Means ± SEM from *n* = 3 independent biological repeats. (C) PI uptake into RPE cells in the presence or absence of YVAD and infected with EPEC-1. Means ± SEM from *n* = 3 independent biological repeats. (D) PI uptake into RPE cells infected with EPEC-1 in complete DMEM or Ca^2+^-free DMEM. Means ± SEM from *n* = 3 independent biological repeats. Statistical significance was determined using 1-way ANOVA with Tukey posttest (B) or 2-tailed *t* test (C, D) ns, nonsignificant; * *p* ≤ 0.05; ** *p* ≤ 0.01. The underlying data for this figure can be found in S1 Data. ANOVA, analysis of variance; DMEM, Dulbecco’s Modified Eagle’s medium; EGTA, ethylene glycol-bis(β-aminoethyl ether)-N,N,N′,N′-tetraacetic acid; EPEC, enteropathogenic *Escherichia coli*; PI, propidium iodide; SEM, standard error of the mean; Tir, translocated intimin receptor; YVAD, z-YVAD-fmk.

To determine if Tir-induced cell death is restricted to tumorigenic epithelial cells, we proceeded to use the primary human retinal pigment epithelium (RPE) cell, a non-tumorigenic model cell line that has been previously used in epithelial cell death studies, including caspase-4-dependent cell death [61]. Infection of RPE cells by EPEC WT and EPEC-1, but not EPEC-0, showed actin pedestal formation, confirming EPEC attachment (Fig 7A). In IFNγ-primed RPE cells, EPEC-1 induced a significantly higher level of cell death compared with both EPEC WT and EPEC-0 (Fig 7B). Similar to SNU-C5, cell death induced by EPEC-1 in RPE cells was inhibited by the pyroptosis inhibitor YVAD as well as by the chelation of extracellular Ca^2+^ (Fig 7C and 7D). This suggests that the ability of Tir to induce Ca^2+^-dependent pyroptosis is conserved across the tumorigenic and primary epithelial cell lines.

### Tir induces Ca^2+^-dependent LPS internalisation

**Fig 8:**
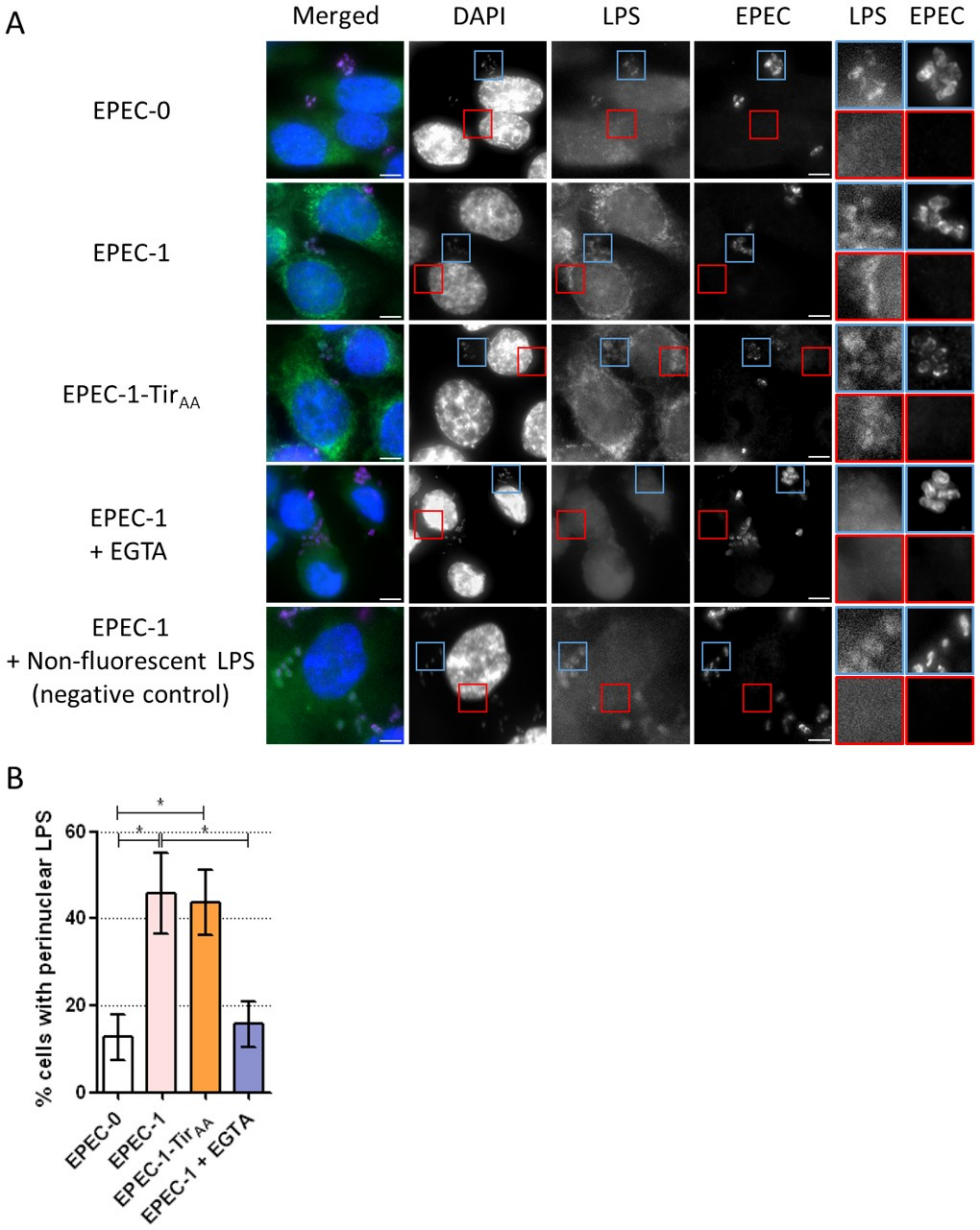
Tir promotes Ca^2+^-dependent LPS internalisation. (A) Alexa-488 conjugated LPS (or non-fluorescent LPS as negative control for autofluorescence) and EGTA was added to primed SNU-C5 cells 30 min before infection. Cells were infected by EPEC-0, EPEC-1 and EPEC-1-Tir_AA_ for 2 h. DAPI: blue; LPS: green; EPEC: purple. Representative images from *n* = 4 biological repeats are shown. Scale bar: 5 μm. Enlarged images of the bacteria (autofluorescence) and perinuclear regions of infected cells are shown with blue and red borders, respectively. (B) The percentage of cells with perinuclear LPS clusters. Means ± SEM from *n* = 4 independent biological repeats are shown. Statistical significance was determined using 1-way ANOVA with Tukey posttest. * *p* ≤ 0.05. The underlying data for this figure can be found in S1 Data. ANOVA, analysis of variance; DAPI, 4′,6-diamidino-2-phenylindole; EGTA, ethylene glycol-bis(β-aminoethyl ether)-N,N,N′,N′-tetraacetic acid; EPEC, enteropathogenic *Escherichia coli*; LPS, lipopolysaccharide; SEM, standard error of the mean; Tir, translocated intimin receptor.

Our data thus far show that Tir-induced cell death is dependent on Ca^2+^ influx and caspase-4 activation. In order to mechanistically integrate these observations, we investigated if Ca^2+^ influx could promote LPS internalisation. To this end, *E*. *coli* LPS conjugated with Alexa-488 was added as a tracer before infection. Fluorescent LPS was detected as clusters surrounding the perinuclear region in approximately a third of EPEC-1- and EPEC-1-Tir_AA_-infected cells, which was significantly higher than EPEC-0-infected cells (Fig 8A and 8B), suggesting that Tir promotes the internalisation of LPS independent of actin polymerisation. Depletion of extracellular Ca^2+^ reduced LPS perinuclear localisation (Fig 8A), indicating the role of Ca^2+^ in the LPS internalisation process. The requirement of both Ca^2+^ and Tir for LPS internalisation mirrors the Tir-induced cell phenotype. Taken together, these results show a novel cascade leading to EPEC-induced cell death, starting with Tir translocation and clustering and induction of Ca^2+^ influx, which promote LPS internalisation and activation of caspase-4.

## Discussion

In this study, we investigate if EPEC can trigger cell death in IECs. We show that similar to macrophages (both THP1 and primary human monocyte-derived macrophages) [25], infection of SNU-C5 cells induced PI uptake that was dependent on Tir, with the magnitude of cell death directly correlated with the extent of Tir translocation. Importantly, in contrast to macrophages, EPEC-induced cell death in IECs was independent of both actin polymerisation and the canonical inflammasome (i.e., NLRP3 and caspase-1). This study provides the first evidence for caspase-4-dependent pyroptosis in the absences of NLRP3 and caspase-1 in IECs upon EPEC infection.

The cell death of SNU-C5 was independent of caspase-8 or caspase-5 (which was undetectable) or necroptosis but, similar to macrophages [25], dependent on caspase-4 and GSDMD. Tir-induced pyroptosis was enhanced by IFNγ, which triggered expression of caspase-4 and GSDMD in SNU-C5, while cell lines endogenously expressing low level of GSDMD were refractory to EPEC-induced cell death. These results demonstrate not only that the genetic background of host cells affects susceptibility to infection, but also that GSDMD is the rate limiting factor impacting on EPEC-induced cell death.

EPEC-induced cell death is not limited to tumorigenic cells, as the primary cell line RPE showed similar susceptibility. Induction of pyroptosis during EPEC infection could benefit the host, by means of secretion of pro-inflammatory cytokines (e.g., IL-18) [11] and specific elimination of infected cells, as cell death is contact dependent (i.e., intimin–Tir interactions). Indeed, the EPEC-like murine pathogen *Citrobacter rodentium* [62,63] lacking EspZ is highly attenuated [64], which is consistent with our findings that uncontrolled Tir translocation leads to removal of infected cells by pyroptosis. On the pathogen side, as intimin–Tir interactions, which are essential for colonisation, lead to unintentional cell death, EPEC injects NleF, an effector expressed in the majority of typical EPEC strains [65], to mitigate the impact by inhibiting caspase-4 and blocking pyroptosis. NleF was also found to be associated more frequently with diarrhoeagenic cases compared with asymptomatic cases in atypical EPEC isolates recently found in Brazil [66], indicating the importance of cell death inhibition in bacterial virulence. Moreover, our data suggest that Tir-induced actin polymerisation also contributes to the moderation of cell death in IECs. This would benefit the pathogen as it could preserve the colonisation site and sustain the infection.

To mechanistically link plasma membrane-associated Tir with cytosolic caspase-4, we performed proteomic analysis to identify proteins and pathways correlated with the cell death levels induced by the different EPEC variants. This revealed the remodelling of diverse cellular processes upon IFNγ treatment and EPEC infection. Several pathways and proteins strongly correlated with cell death levels were induced by the different EPEC strains. Others showed a negative correlation with cell death level, including cell cycle, cell division, and protein degradation machinery, which have also been demonstrated in previous researches to be released by pyroptotic cells [67]. Notably, Ca^2+^-related cellular processes, including activation of the transcription factor CREB1, clearly correlated with the cell death gradient. While infection resulted in higher abundance of Ca^2+^ transporters, the precise route of Ca^2+^ influx remains unclear. The rapid onset of Ca^2+^ influx independent of priming suggests the involvement of an IFNγ-independent posttranslational mechanism. Intimin-mediated Tir clustering has been shown to cause plasma membrane curvature, resulting in localised membrane disruption that could facilitate the influx of Ca^2+^. This hypothesis is consistent with the correlation between the level of Tir translocation and the magnitude of pyroptosis. Tir clustering may trigger the activation of Ca^2+^ channels in the plasma membrane. For example, members of the transient receptor potential (TRP) cation channel family that responds to mechanical changes in the local membrane [68,69] could be activated by such stimulus. In addition, *E*. *coli* LPS can intercalate into the plasma membrane leading to membrane compression which activates certain members of the TRP family [69–72]. TRPV2 and TRPM7, expressed in SNU-C5 [39], can facilitate Ca^2+^ influx in response to both membrane stretch [73–75] and LPS exposure [76,77]. The ability of TRPV2 to promote LPS-dependent NF-κB activation [76] also mirrors our finding during EPEC-1 and EPEC-1-Tir_AA_ infection.

While extracellular Ca^2+^ is essential for Tir-induced pyroptosis, it is dispensable for cell death by caspase-4 in another scenario, for example, the delivery of LPS by transfection. Indeed, our results indicate that the role of Ca^2+^ influx is in promoting LPS internalisation. Increased intracellular Ca^2+^ concentration can activate diverse cell signalling events, while Ca^2+^ overload in mitochondria can lead to opening of the mitochondrial permeability transition pore, release of cytochrome c, and ROS production [58,78]. However, we have shown that neither ROS nor apoptosis are involved in EPEC-induced cell death in SNU-C5 cells. While inhibiting Ca^2+^ influx by extracellular Ca^2+^ chelation and growing SNU-C5 cells in Ca^2+^-free medium inhibited Tir-induced cell death, inducing a sharp preinfection Ca^2+^ influx by ATP increased the level of Tir-induced pyroptosis, while not affecting LPS-induced cell death. Together, these findings strongly suggest that Ca^2+^ influx plays a key role upstream of cell death. Notably, Ca^2+^ influx alone did not induce cell death in the absence of Tir, suggesting that Ca^2+^ synergies but does not replace Tir-dependent downstream processes.

Recent studies have shown that *E*. *coli* and *Streptococcus pneumoniae* induce cytosolic Ca^2+^ up-regulation in murine macrophages, which was dependent on the cooperation between the mitochondrial proteins TMEM173 and ITPR1 (the main ER Ca^2+^ release channel) [56]. Moreover, TMEM173-mediated Ca^2+^ release from intracellular stores promoted cleavage of GSDMD by caspase-1/11 or caspase-8 in response to *E*. *coli* or *S*. *pneumoniae*, respectively [56]. Conversely, the overexpression of ATP2A2, which was found to be up-regulated by Tir and predicted to be downstream of CREB1 in our study, limited *E*. *coli* and *S*. *pneumoniae*-induced GSDMD N-terminus fragment formation [56], suggesting the presence of negative feedback during Tir-induced cell death.

Intracellular compartmentalisation of LPS was detected in Tir- and Ca^2+^-dependent manners. LPS uptake has been attributed to multiple plasma membrane proteins, including the LPS-binding protein (LBP) and TLR4 [79,80]. Although TLR4-dependent LPS uptake also requires extracellular Ca^2+^ influx [77], TLR4 is not detected in the SNU-C5 proteome [39]. Scavenger receptor-mediated LPS uptake and the subsequent perinuclear compartmentalisation have been characterised in HeLa cell overexpressing the scavenger receptor class B member 1 (SCARB1) [81]. SCARB1 and the LPS-binding apolipoproteins apolipoprotein B (APOB) and apolipoprotein E (APOE) are up-regulated upon EPEC-1 and EPEC-1-Tir_AA_ infections correlating with the cell death gradient, suggesting a possible role of these proteins in LPS uptake.

**Fig 9:**
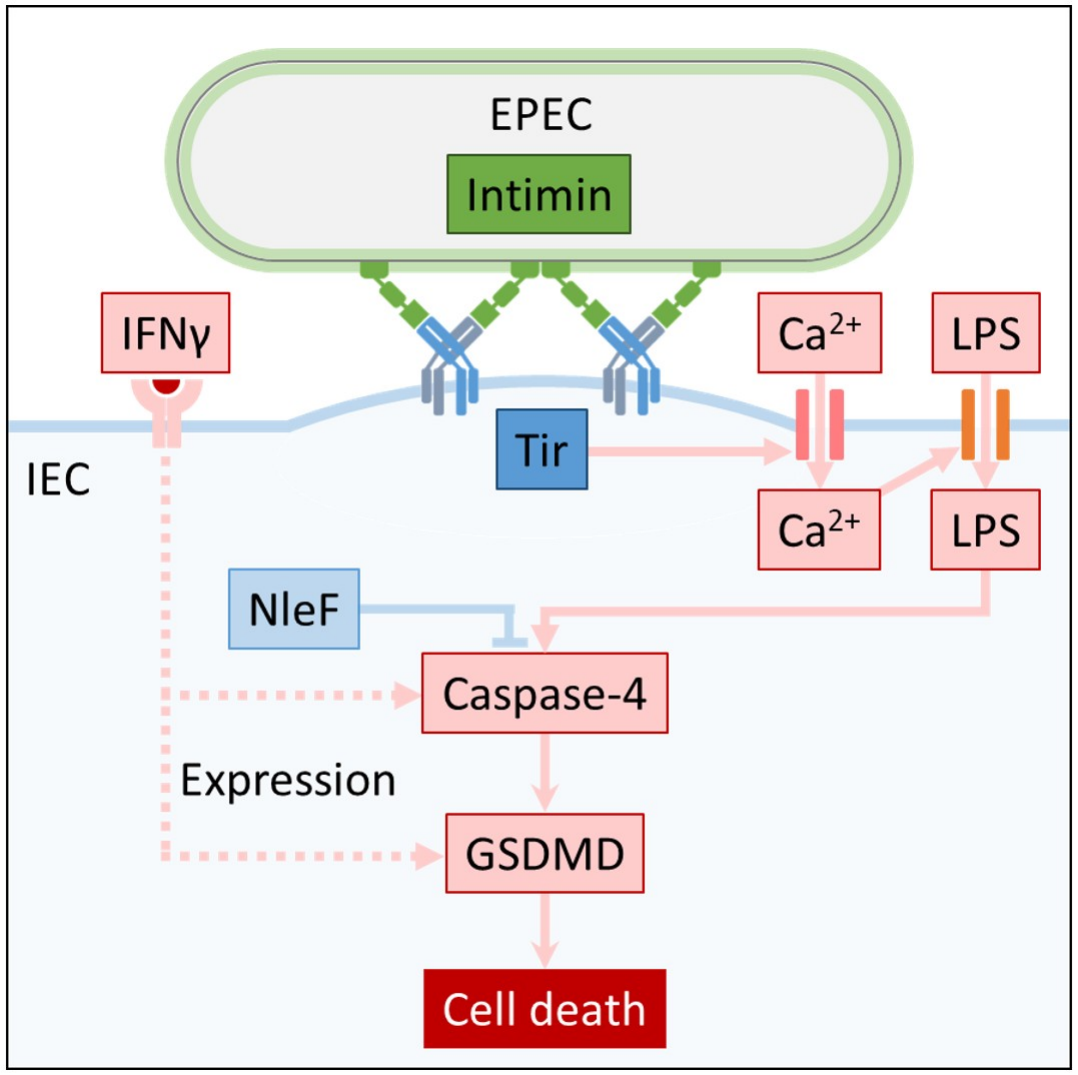
A model portraying the pathway of EPEC-induced cell death. EPEC infection is initiated with Tir translocation. Binding of intimin leads to intimate attachment, Tir clustering, and local membrane curvature, which could activate Ca^2+^ transporters and influx. Ca^2+^ influx leads to LPS entry, which can lead to the activation of caspase-4 and pyroptosis, while NleF can inhibit caspase-4 activation. IFNγ priming induces expression of caspase-4 and GSDMD, enhancing cell death. EPEC, enteropathogenic *Escherichia coli*; GSDMD, gasdermin D; IFNγ, interferon gamma; LPS, lipopolysaccharide; Tir, translocated intimin receptor.

Intimin-mediated Tir clustering has been shown to cause plasma membrane curvature, which could facilitate the influx of both Ca^2+^ and LPS. This hypothesis is consistent with the correlation between the level of Tir translocation and the magnitude of pyroptosis. As Ca^2+^ has been shown to stabilise LPS at the bacterial outer membrane [82,83], it may also facilitate adhesion of free LPS to the plasma membrane of infected cells, which likely increases its chance of entry via Tir-induced local membrane permeability. Overall, our results converge to a novel pathway, which relies on influx of Ca^2+^ through the plasma membrane during infection with an extracellular pathogen, in a Tir-dependent manner, leading to LPS internalisation followed by caspase-4-mediated GSDMD cleavage (Fig 9).

## Materials and methods

### Bacterial strains and cell lines

EPEC E2348/69 strains (S1A Table) were grown in Luria Bertani (LB) (Sigma-Aldrich, St. Louis, Missouri, United States of America) broth or agar. Overnight bacterial cultures were grown at 37°C 180 revolutions per minute (rpm) shaking (liquid) or static (agar) and primed in Dulbecco’s Modified Eagle Medium (DMEM) as described below for infections.

SNU-C5, HT-29, SNU-C2B, COLO-320-HSR, and THP-1 cell lines were cultured in Roswell Park Memorial Institute (RPMI) medium (Sigma-Aldrich) with 10% (v/v) foetal bovine serum (FBS) (Gibco, Carlsbad, California, USA), 2 mM Glutamax (Gibco), 1 mM sodium pyruvate (Sigma-Aldrich), 10 mM N-2-hydroxyethylpiperazine-N-2-ethane sulphonic acid (HEPES) (Sigma-Aldrich), and 2,500 μg/ml glucose (Sigma-Aldrich). THP-1 cells were differentiated using 100 ng/ml phorbol 12-myristate 13-acetate (PMA) (Sigma-Aldrich) for 48 h and PMA withdrawn for 24 h. RPE cell line was cultured in DMEM/F-12 medium (Sigma-Aldrich) with 10% (v/v) FBS, 2 mM Glutamax, and 0.26% sodium bicarbonate (Gibco). HEK293E cell line was cultured in DMEM (high glucose, 4,500 mg/L) (Sigma-Aldrich) with 10% (v/v) FBS, 1 mM sodium pyruvate, and 10 mM HEPES (pH 7.4).

### Generating of EPEC-1-Tir_AA_ strain

Genomic DNA from WT EPEC was extracted using the DNeasy Blood & Tissue Kit (Qiagen, Hilden, Germany). All primers were ordered from Merck (Darmstadt, Germany). Primers 1.1.1 and 1.1.2 were used to amplify *tir* gene from the genomic DNA. The *tir* gene and the pSEVA612S plasmid were digested by *Bam*HI and *Hin*dIII and ligated to make pSEVA612S-*tir* followed by transformation into *E*. *coli* CC118λpir. Primers 1.3.2 and 1.4.2 were used to mutate the Y454 and Y474 in pSEVA612S-*tir* to alanine. The resulting linear PCR product was digested by *Dpn*I and ligated to create pSEVA612S-*tir*_AA_ plasmid and then transformed into *E*. *coli* CC118λpir. Mutations were confirmed by plasmid sequencing (Eurofins Genomics, Constance, Germany) using primers M13-FP, M13-RP, 1.7, and 1.8.

pSEVA612S-*tir*_AA_ plasmid was conjugated from *E*. *coli* CC118λpir pSEVA612S-*tir*_AA_ into EPEC-0 (containing pACBSR plasmid) using the helper strain *E*. *coli* 1047 pRK2013. Colonies that grew on agar plates with both gentamicin and streptomycin were selected, and chromosomal integration of pSEVA612S-*tir*_AA_ was confirmed by colony PCR using primers 1.5 and 1.6. All plasmid descriptions and primer sequences are listed in S1B and S1C Table, respectively.

### Infection of IECs

Cells were seeded 1 day before infection. EPEC strains were primed by diluting the overnight cultures 50× in non-supplemented DMEM (low glucose) and growing for 3 h static at 37°C with 5% CO_2_. Isopropyl β-d-1-thiogalactopyranoside (IPTG) (Sigma-Aldrich) at 1 mM was added to the bacterial culture 30 min before infection when required. Infection was carried out at a multiplicity-of-infection (MOI) of 50:1. Spent medium was replaced with fresh serum-free RPMI (SNU-C5, HT-29, SNU-C2B, COLO-320-HSR and THP-1) or DMEM medium (RPE) 1 h before infection. SNU-C5 and RPE cells for infection in Ca^2+^-free condition were grown either in Ca^2+^-free DMEM (high glucose) (Gibco) (supplemented with 1 mM sodium pyruvate to the same level as Ca^2+^-containing DMEM) or Ca^2+^-containing DMEM (high glucose) (Sigma-Aldrich) as control from 1 h before infection. Infected cells were centrifuged at 700 g for 10 min and incubated for 2 h static at 37°C, 5% CO_2_. For over 2 h of infection, 200 μg/ml gentamicin (Sigma-Aldrich) or 250 μg/ml kanamycin (Sigma-Aldrich) (for EPEC-1-Tir_AA_ strain) was added 2 h postinfection.

### Cytokine and drug treatment

A total of 10 ng/ml human recombinant-IFNγ (R&D Systems, Minneapolis, Minnesota, USA) was added to the cells 24 h before infection. Moreover, 50 μM z-VAD-fmk (zVAD) (R&D Systems), 50 μM z-YVAD-fmk (YVAD) (R&D Systems), 25 μM necrostatin-1 (Nec1) (Santa Cruz Biotechnology, Dallas, Texas, USA), 5 μM necrosulfonamide (NSA) (Tocris), 200 nM cytochalasin D (Sigma-Aldrich), 1 mM EGTA (Sigma-Aldrich), 0.5 mM ATP (Sigma-Aldrich), 5 μM MCC950 (Tocris), 200 μM Tiron (Sigma-Aldrich), 50 mM N,N′-dimethylthiourea (DMTU) (Sigma-Aldrich), 10 μM oligomycin (Sigma-Aldrich) or 2 μM staurosporine (STS) (Calbiochem, San Diego, California, USA) were added to the cells 30 min before infection. For co-treatment, ATP was added 30 min before infection, while EGTA or YVAD were added 45 min before infection. Ultrapure *E*. *coli* O111:B4 LPS (Invivogen, San Diego, California, USA) transfection was performed using Lipofectamine 2000 (Invitrogen, Waltham, Massachusetts, USA) at 5 μg/ml.

### siRNA transfection

A total of 1.25 × 10^4^ cells/well were seeded in black clear-bottom 96-well plates 3 days prior to infection. In addition, 50 mM siRNA (0.25 μl) (Dharmacon, Lafayette, Colorado, USA) (S1D Table) mixed with 0.3 μl of TransIT-X2 transfection reagent (Mirus Bio, Madison, Wisconsin, USA) in 9 μl Opti-MEM were added to the cells 2 days before infection (caspase-4 siRNA) or 6 h after seeding, i.e., 3 days before infection (GSDMD, caspase-8 and RIPK3 siRNA). The medium was replaced with IFNγ-containing RPMI 1 day before infection.

### *GSDMD* stable silencing

Retroviral plasmid pMX-CMV-YFP-LacZ-miRNA30E and pMX-CMV-YFP-GSDMD-miRNA30E (S1B and S1D Table) [25, 50, 84] were packaged in HEK293E cells with the packaging plasmid pCMV-MMLV-Gag/Pol and pCMV-VSV-G, in a 5:4:1 ratio, using Lipofectamine 2000. After 48 h of viral transfection, the medium containing the virus was filtered through a 0.45-μm filter and transferred to pre-seeded SNU-C5 cells. Puromycin (5 μg/ml) (Gibco, Carlsbad, California, USA) was added to the SNU-C5 cells 48 h after viral transduction. Cells were sorted by FACS Aria III Flow Sorter in CMBI high throughput single-cell analysis facility (HTSCAF) for yellow fluorescent protein (YFP) expression. Puromycin treatment was continued after sorting to maintain a stable YFP-expressing population.

### Immunofluorescence staining

A total of 1.5 × 10^5^ cells/well were seeded in 24-well plates on glass coverslips for imaging with Zeiss AxioImager Z1 microscope (Carl Zeiss, Jena, Germany). A total of 1.5 × 10^4^ cells/well were seeded in black clear-bottom 96-well plates for imaging with Opera Cell::Explorer automated spinning-disk confocal microscope (Perkin-Elmer, Waltham, Massachusetts, USA). Infection experiments were carried out as described. Alexa-488-conjugated *E*. *coli* O55:B5 LPS (Thermo Fisher Scientific, Waltham, Massachusetts, USA) was added to the cells at 5 μg/ml 30 min before infection when required. Cells were fixed by 4% paraformaldehyde for 15 min, washed by 3× PBS, permeabilised by 0.2% Triton X-100 (Sigma-Aldrich) for 4 min, washed again and blocked with 1% bovine serum albumin (BSA) for 10 min before being incubated with primary antibodies (S1E Table) for 45 min. Cells were re-blocked with 1% BSA for 10 min and incubated with secondary antibodies (S1E Table) for 30 min. For imaging using Z1 microscope using 100× oil lens, coverslips were mounted on glass slides (VWR, Radnor, Pennsylvania, USA) with Gold-Pro-Long-Anti-fade (Invitrogen).

### Western blotting

Cells were lysed with lysis buffer containing radioimmunoprecipitation (RIPA) buffer (50 mM Tris-HCl, pH 8, 2 mM EDTA, 300 mM NaCl, 2% NP-40, 1% sodium deoxycholate) and 1× protease inhibitor (Pierce, Waltham, Massachusetts, USA) and mixed with 1× Laemmli sample buffer (Bio-Rad, Hercules, California, USA) and 5% β-mercaptoethanol (Sigma-Aldrich). The lysates were run on sodium dodecyl sulphate polyacrylamide gel electrophoresis (SDS-PAGE) gels. Western blotting was performed using a TransBlot Turbo Transfer System (Bio-Rad) to transfer the protein bands to the polyvinylidene difluoride (PVDF) membrane. Membranes were blocked with 5% milk in phosphate buffered saline, 0.05% Tween 20 (PBST) or tris buffered saline, 0.1% Tween 20 (TBST) for 1 h at room temperature and probed with primary antibodies (S1F Table) overnight at 4°C. The secondary antibodies (S1F Table) were added for 1 h at room temperature. Membranes were developed using the ECL Western Blotting Reagents (GE, Amersham, UK) and imaged using the ChemiDoc MP imaging system (Bio-Rad).

### Infection rate measurement

A total of 1.5 × 10^5^ cells/well were seeded in 24-well plates. Infection was carried out 1 day post-seeding as described for 2 h (no antibiotic). Infected cells were washed by 3× PBS and treated by 0.5% porcine pancreas trypsin and 0.02% EDTA in PBS (Sigma-Aldrich) for counting by haemocytometer (Hecht Glaswarenfabrik, Rhön, Germany). Counted cells were lysed with 0.1% Triton X-100, serially diluted and plated. Colony forming units (CFUs) were counted after overnight incubation at 37°C. Infection rate was calculated by CFU per cell.

### High-content image acquisition and analysis

Imaging was performed using an Opera Cell::Explorer automated spinning-disk confocal microscope using 40× lens (Perkin-Elmer). Twenty-five evenly distributed fields were imaged per well. Columbus-2 System (Perkin-Elmer) was used for image analysis. Nucleus and cytosolic segmentation was performed using the DAPI and phalloidin staining, respectively.

NF-κB activation was represented by the p65 nuclear-to-cytosolic ratio measured and calculated by Columbus-2. The nucleus region for p65 intensity measurement was 2 pixels inward from the nuclear–cytosolic boundary defined by DAPI to avoid segmentation errors at the boundary. The cytosolic region was a 2-pixel-ring further 2-pixel-outward from the nuclear–cytosolic boundary to avoid measurement skewing by cell shape [85].

### PI uptake assay

A total of 5 × 10^4^ cells/well were seeded in black clear-bottom 96-well plates 1 day prior to infection and IFNγ and inhibitor treatment were applied as described previously. Alternatively, 1.25 × 10^4^ cells/well were seeded in 3 days prior to infection for siRNA transfection as described before. Bacteria were primed in phenol-red-free DMEM (low glucose). Cells were incubated in phenol-red-free RPMI or DMEM, for different cell lines as described previously, supplemented with 5 μg/ml PI (Sigma-Aldrich) 1 h prior to infection and throughout the infection. Cell-free medium-only wells were prepared as blank. Positive control wells were prepared by cell lysis using RPMI with 5 μg/ml PI and 0.05% Triton X-100 (Sigma-Aldrich) 10 min prior to infection. Infections were carried out as described before. Time course measurements were carried out from 10 min to 8 h postinfection with 10-min intervals, measuring 620-nm emission with 520-nm excitation. The percentage of PI uptake of each well was calculated by dividing each blank-normalised reading over blank-normalised positive control reading at the same time point. The increase in percentage PI uptake of the infected cells compared with uninfected was then calculated by subtraction of the percentage PI uptake in uninfected samples (with the same drug/siRNA treatments).

### qRT-PCR

A total of 5 × 10^5^ cells/well were seeded in 12-well plates 1 day prior to RNA extraction. When required, IFNγ treatments were applied as described previously at the time of seeding. RNA extraction using the RNeasy kit (Qiagen) and DNase digestion using the RQ1 RNase-free DNase (Qiagen) were performed following the manufacturer’s instructions. A total of 0.5 μl each of oligo-dT and random primers (Promega, Madison, Wisconsin, USA) were added to every 1 μg RNA and incubated at 70°C for 5 min. M-MLV reverse transcriptase (Promega) was used to perform the RT reaction at 42°C for 60 min following the manufacturer’s instructions. q-PCR reactions were performed on 10 ng cDNA/sample using gene-specific primers and PowerUp SyBr-Green Mastermix in a StepOne Real-Time PCR system (Thermo). Data analysis was performed using StepOne Software v2.3. Glyceraldehyde 3-phosphate dehydrogenase (GAPDH) cDNA level was used as an internal control. Data used for statistical analysis were then normalised by log 2 transformation of the fold change to untreated control.

### Fluo-4 assay

A total of 5 × 10^4^ cells/well were seeded in black clear-bottom 96-well plates 1 day prior to infection, and IFNγ was applied as described. Cells were incubated in Fluo-4 Direct reagent (Molecular Probes, Eugene, Oregon, USA) diluted 2-fold in phenol-red-free RPMI for 30 min before the first measurement. Fluorescence readings were performed in Omega microplate reader measuring 520-nm emission with 485-nm excitation. All drugs were added 30 min before infection, unless specified otherwise. ATP-treated samples were followed by a second measurement at 2 min after treatment. Cell-free medium-only wells were prepared as blank. When required, time course measurements were carried out from 20 min before to 1 h after infection with 10-min intervals. Raw measurements of Fluo-4 fluorescence of each well were normalised by subtraction of the fluorescence of the blank well.

### Agilent Seahorse metabolic assays

A total 1.5 × 10^4^ cells/well were seeded in XFp Cell Culture Miniplates (Agilent Technologies, Santa Clara, California, USA) 1 day prior to measurement. Cells were incubated at 37°C in non-CO_2_ incubator for 45 min in XF RPMI base medium with 2 mM glutamine, 1 mM sodium pyruvate, and 10 mM glucose, with pH 7.4. Measurements of oxygen consumption rate (OCR) were carried out in a Seahorse XFp Analyzer (Agilent Technologies). After measurements, OCR was normalised by the number of cells counted by haemocytometer after trypsin digestion.

### Sample preparation and TMT labelling

Cell pellets were solubilised in lysis buffer (100 mM triethylammonium bicarbonate (TEAB), 1% sodium deoxycholate (SDC), 10% isopropanol, 50 mM NaCl) supplemented with halt protease and phosphatase inhibitor cocktail (Thermo Fisher Scientific, Catalog Number 78442), assisted with pulsed probe sonication. The samples were subsequently boiled for 5 min at 90°C and then subjected to a second round of sonication. The protein concentration was measured with the Coomasie Plus assay (Thermo Fisher Scientific) according to manufacturer’s instructions. Aliquots containing 100 μg of protein were prepared for trypsin digestion. Samples were reduced with 5 mM tris-2-carboxyethyl phosphine (TCEP) and alkylated with 10 mM iodoacetamide (IAA). Proteins were then digested by adding trypsin at the 75 ng/μl final concentration and incubating the samples for 18 h at room temperature, shaking at 300 rpm. The resultant peptides were diluted up to 100 μl with 100 mM TEAB buffer and labelled with tandem mass tags (TMTs; TMT11plex) multiplex reagents (Thermo Fisher Scientific) according to manufacturer’s instructions. The quenching reaction was performed by adding hydroxylamine to a final concentration of 0.27% (v/v) and incubating the samples for 15 min at room temperature. Finally, 11 samples per batch were combined in equal amounts to a single tube. Formic acid was added to a final concentration of 2% (v/v), and samples were centrifuged for 5 min at 10,0000 rpm to precipitate SDC. The supernatant containing TMT-labelled peptides was moved to a new tube and dried with a centrifugal vacuum concentrator.

### High-pH reversed-phase peptide fractionation and LC–MS analysis

Offline high-pH reversed-phase (RP) peptide fractionation and desalting were performed using the Waters (Milford, Massachusetts, USA) XBridge C18 column (2.1 × 150 mm, 3.5 mm) on a Dionex Ultimate 3000 (Thermo Fisher Scientific) high-performance liquid chromatograph (HPLC) system. Mobile phase A was 0.1% ammonium hydroxide, and mobile phase B was acetonitrile and 0.1% ammonium hydroxide. The TMT-labelled peptides were reconstituted in 100 μl of mobile phase A and fractionated using a multistep gradient elution method at 200 μl/min as follows: 5 min at 5% phase B, for 35 min up to 35% phase B, up to 80% phase B in 5 min, isocratic for 5 min and re-equilibration to 5% phase B. Fractions were collected in a 96-well plate every 42 s to a total of 65 fractions, then concatenated into 28 fractions and vacuum dried. Liquid chromatography–mass spectrometry (LC–MS) analysis was performed on the Dionex Ultimate 3000 system coupled with the Orbitrap Fusion Lumos mass spectrometer (Thermo Fisher Scientific). Each peptide fraction was reconstituted in 50 μl 0.1% formic acid, and 10 μl were loaded to the Acclaim PepMap 100, 100 μm × 2 cm, 5 μm, 100 Å C18 trapping column (Thermo Fisher Scientific) at a 10 μl/min flow rate. The sample was then analysed with the EASY-Spray C18 capillary column (75 μm × 50 cm, 2 μm) (Thermo Fisher Scientific) at 45°C. Mobile phase A was 0.1% formic acid, and mobile phase B was 80% acetonitrile and 0.1% formic acid. The gradient separation at a flow rate of 300 nl/min was gradient for 90 min from 5% to 38% phase B and for 10 min up to 95% phase B, isocratic for 5 min at 95% B, re-equilibrated to 5% phase B in 5 min, and isocratic for 10 min at 5% phase B. Precursors between 375 and 1,500 m/z were selected with mass resolution of 120,000, automatic gain control (AGC) of 4 × 10^5^, and injection time (IT) of 50 ms, with the top speed mode in 3 s, and were isolated for collision-induced dissociation (CID) fragmentation with a quadrupole isolation width of 0.7 Th (Thomson unit). Collision energy was set at 35%, with AGC at 1 × 10^4^ and IT at 50 ms. MS3 quantification was obtained with higher-energy collisional dissociation (HCD) fragmentation of the top 5 most abundant CID fragments isolated with synchronous precursor selection (SPS). Quadrupole isolation width was set at 0.7 Th, collision energy was applied at 65%, and the AGC setting was at 1 × 10^5^ with IT at 105 ms. The HCD MS3 spectra were acquired for the mass range of 100 to 500 m/z with a resolution of 50,000. Targeted precursors were dynamically excluded for further isolation and activation for 45 s with 7 ppm mass tolerance. The raw mass spectrometry files have been deposited to PRoteomics IDEntifications database (PRIDE accession: PXD018763).

### Database search and protein quantification

The acquired mass spectra were searched for protein identification and quantification in Proteome Discoverer 2.3 (Thermo Fisher Scientific) with the SEQUEST-HT search engine. The spectra were searched for fully tryptic peptides with maximum of 2 missed-cleavages, 20 ppm precursor mass tolerance, and 0.5 Da fragment ion mass tolerance. The search considered static carbamidomethylation of Cys residues and TMT6plex modification of peptide N-term and Lys residues, as well as dynamic oxidation of Met and deamidation of Asp and Glu residues. The Percolator node was used to estimate the confidence of peptide identifications at 1% false discovery rate (FDR) and was based on the q-value and a decoy database search. The spectra were searched against UniProt annotated reference proteomes of Homo sapiens and *E*. *coli*. The reporter ion quantifier node included a TMT-11-plex quantification method with an integration window tolerance of 15 ppm and integration method based on the most confident centroid peak at MS3 level. Quantification was performed using unique peptides only, with protein groups considered for peptide uniqueness. Proteomics data used for statistical analysis were scaled and then normalised by log 2 transformation of the fold change to uninfected control.

### Statistical analysis

All experiments except proteomics were independently repeated at least 3 times as indicated in the figure legends. Proteomics were independently repeated twice. For qRT-PCR, high-content imaging analysis and CFU assays, 2 to 3 technical repeats were performed to calculate means for each biological repeat. For manual cell counting in microscopy images, at least 4 images from randomly selected positions in each coverslip containing a total of 50 to 100 cells (over 250 cells from 4 biological repeats) were used. For high-content image analysis, 25 images evenly distributed in predefined positions containing a total of over 100 cells in each well were used. Methods of data transformation are described in their corresponding method sections.

Statistical analysis of all biochemical/biological experimental data was performed using GraphPad Prism 5.1. Student *t* test and 1-way or 2-way ANOVA followed by Tukey posttest or Bonferroni posttest, respectively, were performed on the means as listed in the figure legends. Significant result was defined as having a *p*-value of <0.05.

Statistical analysis, including PCA, ANOVA, *t* test, and pathway enrichment analysis of the proteomic data, was performed in Perseus 1.6.2.2 (Max Planck Institute of Biochemistry, Germany) [86]. Pathway enrichment was performed using Uniprot Keywords, Gene Ontology Biological Process (GOBP) and KEGG pathways [87]. For transcription factor analysis, the “TF-LOF Expression from GEO” library was downloaded from the Enrichr web tool [88] and was converted to Perseus-compatible annotation file for 1D annotation enrichment analysis. All biological terms were filtered for Benjamini–Hochberg FDR < 0.05. Heatmaps were plotted in Phantasus (https://artyomovlab.wustl.edu/phantasus/), and boxplots were plotted in R-Studio.

## Supporting information

S1 Data Excel spreadsheet containing the numerical data used for the main and Supporting information figures. (XLSX)

S1 Raw Images Raw images of the western blots presented in Fig 2E, 2F and 2I–2K and S2A, S2G and S2I Fig. (PDF)

S1 Fig Kinetics of Tir-induced cell death throughout 8 h of infection. (A) *Gbp2* expression level was measured by qRT-PCR in IFNγ-primed cells and normalised to the *Gbp2* expression level in the untreated cells. Means ± SEM from *n* = 3 independent biological repeats. Statistical significance was determined using 2-tailed *t* test. * *p* ≤ 0.05. (B) PI uptake into unprimed SNU-C5 cells infected with EPEC WT, EPEC-0 and EPEC-1. Measurements were taken every 10 min. The time-course PI uptake was plotted. PI uptake results were normalised by UI. Means ± SEM from *n* = 5 independent biological repeats are shown. (C) PI uptake into IFNγ-primed uninfected cells. PI uptake results were normalised to unprimed UI cells. Means ± SEM from *n* = 7 independent biological repeats are shown. The underlying data for this figure can be found in S1 Data. EPEC, enteropathogenic *Escherichia coli*; IFNγ, interferon gamma; PI, propidium iodide; qRT-PCR, real-time quantitative PCR; SEM, standard error of the mean; Tir, translocated intimin receptor; UI, uninfected; WT, wild-type.
(TIF)

S2 Fig Apoptosis, NLRP3-dependent pyroptosis and necroptosis do not occur during EPEC-1 infection. (A) Cell lysates of primed SNU-C5 cells infected with EPEC WT, EPEC-0, and EPEC-1, or treated by STS for 20 h, were used for PARP1 western blot. Representative blots were shown. (B) PI uptake into THP1 cells treated by LPS for 3 h followed by nigericin for 8 h, with or without MCC950 treatment 30 min before nigericin addition. Means ± SEM from *n* = 3 independent biological repeats. (C) PI uptake into primed SNU-C5 cells infected with EPEC-1 treated with MCC950 30 min before infection. Means ± SEM from *n* = 3 independent biological repeats. (D, E) PI uptake into SNU-C5 cells treated by YVAD, Nec1 and NSA 30 min before LPS transfection (D) or STS and zVAD treatment (E). Means ± SEM from *n* = 3 independent biological repeats. (F) PI uptake into SNU-C5 cells infected with EPEC-1 treated with Nec1, NSA, zVAD, and a combination of zVAD, Nec1, and NSA 30 min before infection. Means ± SEM from *n* = 3 independent biological repeats. (G) Caspase-8 western blot of SNU-C5 cells transfected with caspase-8 siRNA. Representative blot from *n* = 3 independent biological repeats are shown. (H) PI uptake into SNU-C5 cells transfected by caspase-4, GSDMD or caspase-8 siRNA or combinations of them and infected with EPEC-1. Means ± SEM from *n* = 3 independent biological repeats. (I) RIPK3 western blot of SNU-C5 cells transfected with RIPK3 siRNA. Representative blot from *n* = 3 independent biological repeats are shown. (J) PI uptake into SNU-C5 cells transfected by RIPK3 siRNA treated by STS and zVAD. Means ± SEM from *n* = 3 independent biological repeats. (K) PI uptake into SNU-C5 cells transfected by caspase-4, GSDMD or RIPK3 siRNA or combinations of them and infected with EPEC-1. Means ± SEM from *n* = 3 independent biological repeats. Statistical significance was determined using 2-tailed *t* test (B, C, J) and 1-way ANOVA with Tukey posttest (D, E, F, H, K). * *p* ≤ 0.05; ** *p* ≤ 0.01; *** *p* ≤ 0.001. The underlying data for this figure can be found in S1 Data. ANOVA, analysis of variance; EPEC, enteropathogenic *Escherichia coli*; GSDMD, gasdermin D; LPS, lipopolysaccharide; NLRP3, NLR family pyrin domain containing 3; NSA, necrosulfonamide; PARP1, poly [ADP-ribose] polymerase 1; PI, propidium iodide; SEM, standard error of the mean; siRNA, small interfering RNA; STS, staurosporine; WT, wild-type; YVAD, z-YVAD-fmk; zVAD, z-VAD-fmk.
(TIF)

S3 Fig EPEC adheres to and Tir induces actin polymerisation in SNU-C5, HT-29, SNU-C2B and COLO-320-HSR. Immunofluorescence staining of SNU-C5, HT-29, SNU-C2B and COLO-320-HSR cells were infected with EPEC-0 and EPEC-1 for 4 h. DAPI: blue; EPEC: green; Phalloidin: red. Representative images from *n* = 3 independent biological repeats are shown. Error bar: 5 μm. The underlying data for this figure can be found in S1 Data. EPEC, enteropathogenic *Escherichia coli*; Tir, translocated intimin receptor.
(TIF)

S4 Fig Tir-dependent cell death in unprimed SNU-C5 is inhibited by YVAD and EGTA. (A, B) PI uptake into unprimed SNU-C5 cells infected with EPEC-1 with 30 min pretreatment with YVAD (A) or EGTA (B). Means ± SEM from *n* = 3 independent biological repeats. (C) PI uptake into unprimed SNU-C5 cells infected with EPEC-0, EPEC-2, EPEC-1-Tir_AA_-EspZ, EPEC-1 and EPEC-1-Tir_AA_. Means ± SEM from *n* = 5 independent biological. Statistical significance was determined using 2-tailed *t* test (A, B) and 1-way ANOVA with Tukey posttest (C). * *p* ≤ 0.05; ** *p* ≤ 0.01; *** *p* ≤ 0.001. The underlying data for this figure can be found in S1 Data. ANOVA, analysis of variance; EPEC, enteropathogenic *Escherichia coli*; PI, propidium iodide; SEM, standard error of the mean;Tir, translocated intimin receptor; YVAD, z-YVAD-fmk.
(TIF)

S5 Fig Illustration of the proteomics workflow used in this study. (A) IFNγ-primed and unprimed SNU-C5 cells were infected with the indicated EPEC strains for 2 h. Extracted proteins were digested with trypsin and peptides were labelled with the TMT 11plex reagents in 2 separate replicate batches. TMT-labelled peptides were subjected to offline high-pH reversed-phase fractionation followed by LC–MS analysis. (B) PCA of proteomics data. EPEC, enteropathogenic *Escherichia coli*; IFNγ, interferon gamma; LC–MS, liquid chromatography–mass spectrometry; PCA, principal component analysis; TMT, tandem mass tag.
(TIF)

S6 Fig Proteomic differences of IFNγ-primed and unprimed cells. Heatmap of the differentially regulated proteins between IFNγ-primed and unprimed SNU-C5 cells (*t* test, FDR < 0.05, absolute log_2_ ratio versus uninfected > 0.5). The underlying data for this figure can be found in S1 Data. FDR, false discovery rate; IFNγ, interferon gamma.
(TIF)

S7 Fig Kinetics of Tir-induced Ca^2+^ influx over 1 h of infection. Fluo-4 assay performed on primed (A, C) and unprimed (B) SNU-C5 cells infected with EPEC-0 (A, B), EPEC-1 (A-C) and EPEC-1-Tir_AA_ (A, B) with or without 30 min pretreatment with EGTA (A, B) and YVAD (C). Means ± SEM from *n* = 3 independent biological repeats. The underlying data for this figure can be found in S1 Data. EPEC, enteropathogenic *Escherichia coli*; SEM, standard error of the mean; Tir, translocated intimin receptor; YVAD, z-YVAD-fmk.
(TIF)

S8 Fig EPEC-1 induces cell death independent of ROS- or mitochondrial respiration-dependent cell death. (A–C) PI uptake into primed SNU-C5 cells infected with EPEC-1, with 30 min pretreatment with DMTU (A), Tiron (B) or oligomycin (C). Means ± SEM from *n* = 3 independent biological. (D) OCR measurement was performed on SNU-C5 cells before or after oligomycin treatment. Means ± SEM from *n* = 3 independent biological repeats are shown. Statistical significance was determined using 2-tailed *t* test. ns, nonsignificant; * *p* ≤ 0.05; ** *p* ≤ 0.01; *** *p* ≤ 0.001. The underlying data for this figure can be found in S1 Data. DMTU, N,N′-dimethylthiourea; EPEC, enteropathogenic *Escherichia coli*; OCR, oxygen consumption rate; PI, propidium iodide; SEM, standard error of the mean.
(TIF)

S1 Table Lists of strains, plasmids, oligonucleotide sequences, and antibodies used in this study. (A) Bacterial strains. (B) Plasmids. (C) Primer sequences. (D) siRNA and miRNA30E sequences. (E) Immunofluorescence antibodies and reagents. (F) Western blot antibodies. siRNA, small interfering RNA.
(DOCX)

## Abbreviations

A/E: attaching and effacing
AGC: automatic gain control
ANOVA: analysis of variance
APOB: apolipoprotein B
APOE: apolipoprotein E
ASC: apoptosis-associated speck-like protein containing a CARD
BSA: bovine serum albumin
CANX: calnexin
CASP4: caspase-4
CFU: colony forming unit
CID: collision-induced dissociation
CREB1: cAMP responsive element binding protein 1
DAMP: damage-associated molecular pattern
DMEM: Dulbecco’s Modified Eagle Medium
DMTU: N,N′-dimethylthiourea
EPEC: enteropathogenic *Escherichia coli*
ER: endoplasmic reticulum
FBS: foetal bovine serum
FDR: false discovery rate
GAPDH: glyceraldehyde 3-phosphate dehydrogenase
GBP2: guanylate-binding protein 2
GEO: Gene Expression Omnibus
GOBP: Gene Ontology Biological Process
GSDMD: gasdermin D
GSDME: gasdermin E
HCD: higher-energy collisional dissociation
HEPES: N-2-hydroxyethylpiperazine-N-2-ethane sulphonic acid
HPLC: high-performance liquid chromatograph
IAA: iodoacetamide
IEC: intestinal epithelial cell
IFNGR: interferon gamma receptor
IFNγ: interferon gamma
IL: interleukin
IPTG: isopropyl β-d-1-thiogalactopyranoside
IRF: interferon regulatory factor
IT: injection time
ITPR2: inositol 1,4,5-trisphosphate receptor type 2
JAK: Janus kinase
JAK-STAT: Janus kinase/signal transducers and activators of transcription
LBP: LPS-binding protein
LC–MS: liquid chromatography–mass spectrometry
LPS: lipopolysaccharide
MOI: multiplicity-of-infection
Nec1: necrostatin-1
NF-κB: nuclear factor kappa B
NLR: NOD leucine-rich repeat protein
NLRP3: NLR family pyrin domain containing 3
NOD: nucleotide-binding oligomerisation domain
NSA: necrosulfonamide
OCR: oxygen consumption rate
OMV: outer membrane vesicle
PAMP: pathogen-associated molecular pattern
PARP1: poly [ADP-ribose] polymerase 1
PBS: phosphate buffered saline
PBST: phosphate buffered saline, 0.05% Tween 20
PCA: principal component analysis
PI: propidium iodide
PI3K: phosphatidylinositol 3-kinase
PMA: phorbol 12-myristate 13-acetate
PRIDE: PRoteomics IDEntifications database
PVDF: polyvinylidene difluoride
qRT-PCR: real-time quantitative PCR
RIPA: radioimmunoprecipitation
RIPK: receptor-interacting serine/threonine-protein kinase
ROS: reactive oxygen species
RP: reversed-phase
RPE: retinal pigment epithelium
rpm: revolutions per minute
RPMI: Roswell Park Memorial Institute
SCARB1: scavenger receptor class B member
SDC: sodium deoxycholate
SDS-PAGE: sodium dodecyl sulphate polyacrylamide gel electrophoresis
SERCA: sarco/endoplasmic reticulum Ca^2+^
shRNA: short-hairpin RNA
siRNA: small interfering RNA
SPS: synchronous precursor selection
STAT1: signal transducer and activator of transcription 1
STING: stimulator of interferon genes
STS: staurosporine
T3SS: type III secretion system
TBST: tris buffered saline, 0.1% Tween 20
TCEP: tris-2-carboxyethyl phosphine
TEAB: triethylammonium bicarbonate
Th: Thomson unit
Tir: translocated intimin receptor
TMEM173: transmembrane protein 173
TMT: tandem mass tag
TRP: transient receptor potential
WGCNA: weighted correlation network analysis
WT: wild-type
YFP: yellow fluorescent protein
