# Supplementary figures and images for: Clustering of Tir during enteropathogenic E. coli infection triggers calcium influx–dependent pyroptosis in intestinal epithelial cells
Source: PLoS Biol. 2020 Dec 30;18(12):e3000986. doi: 10.1371/journal.pbio.3000986 (PMC7773185; doi:10.1371/journal.pbio.3000986)

2E

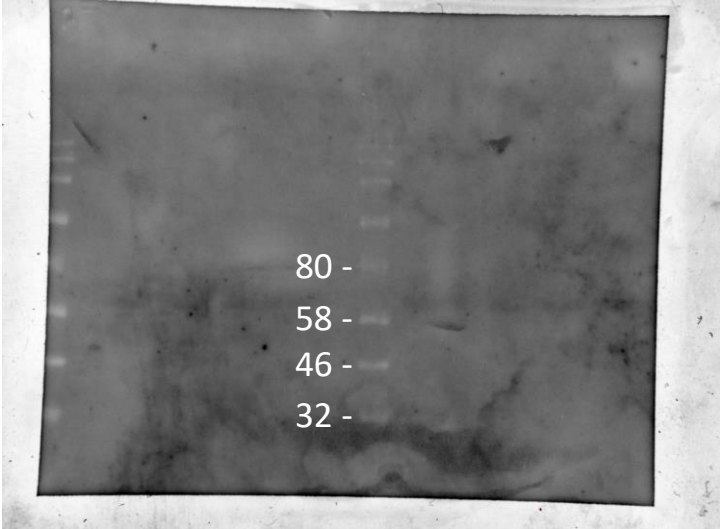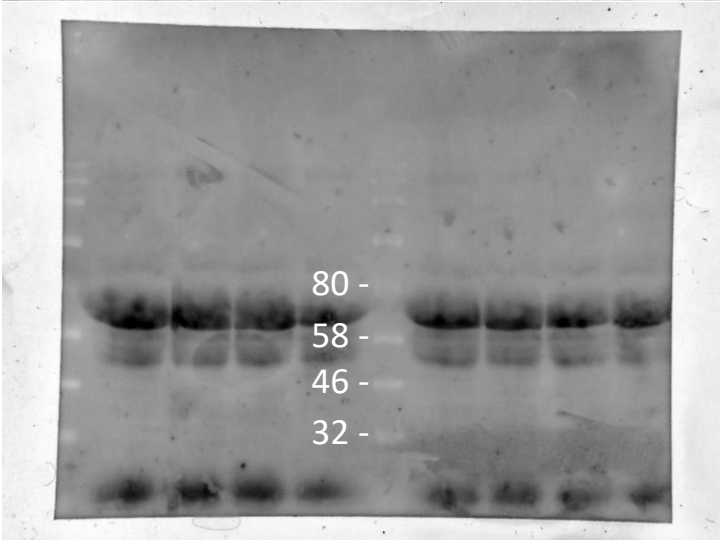

Caspase-4

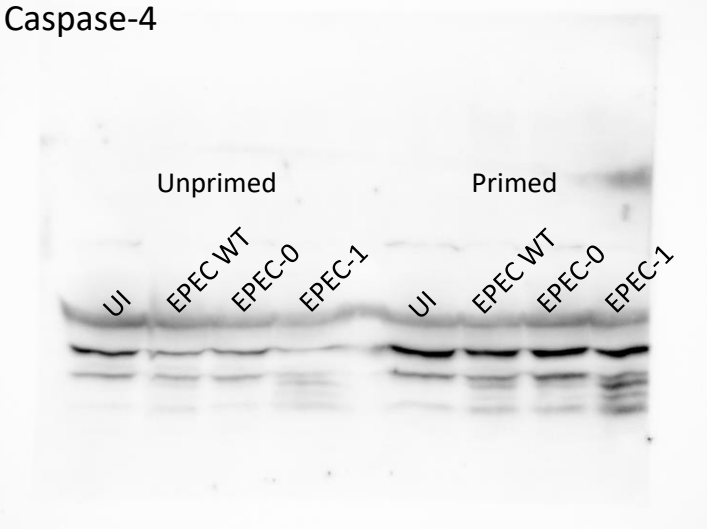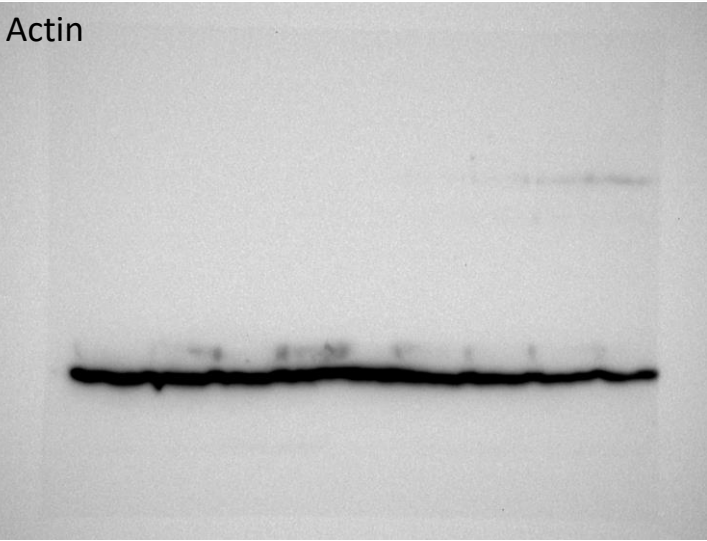

2F

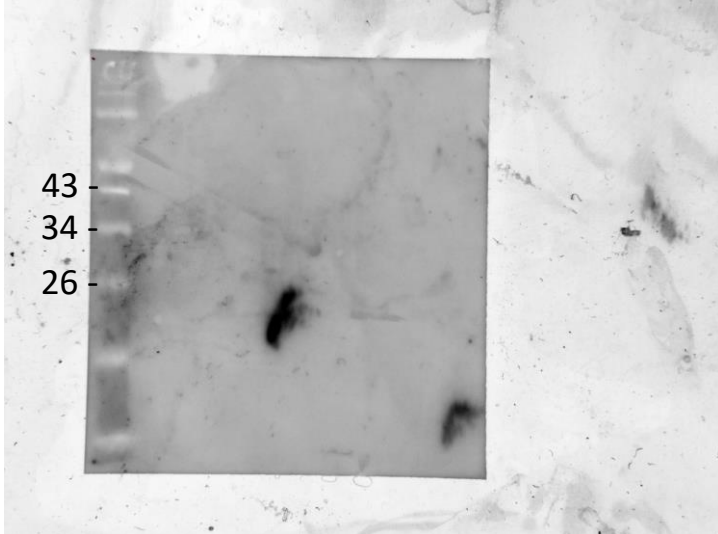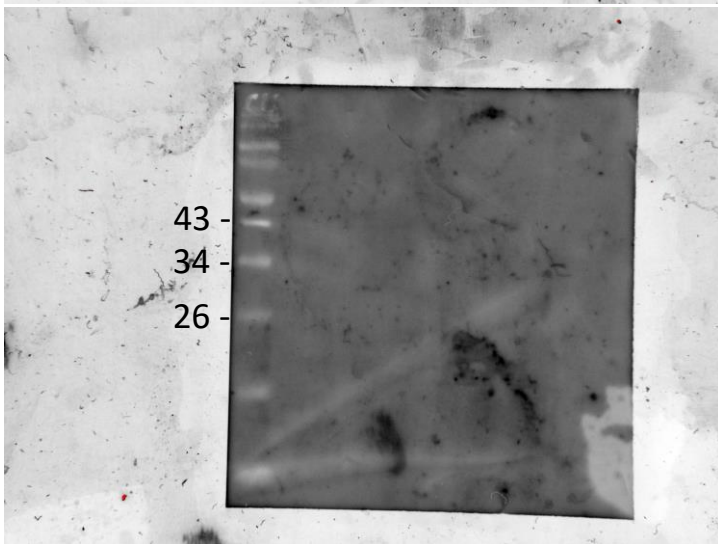

Caspase-4

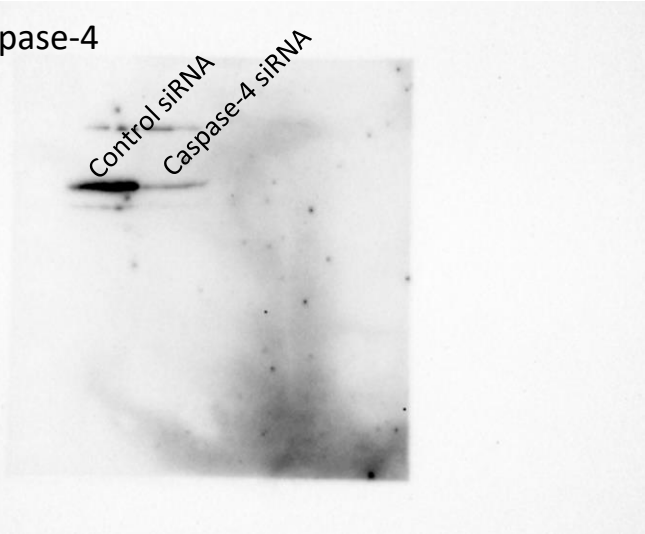

Actin

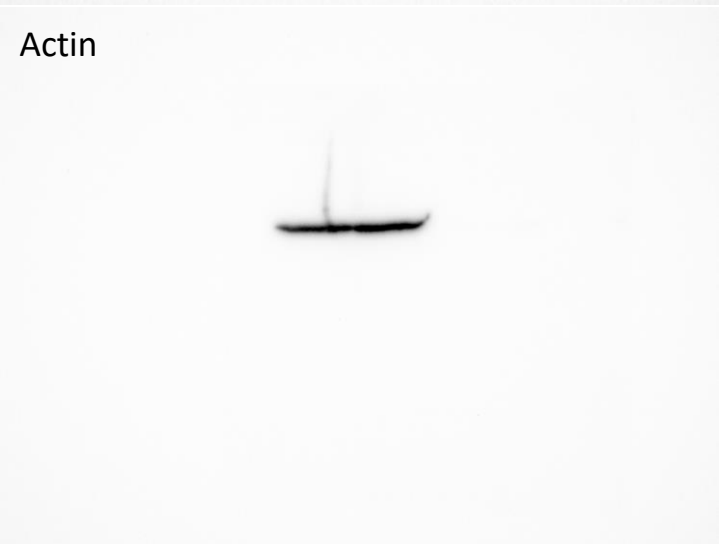

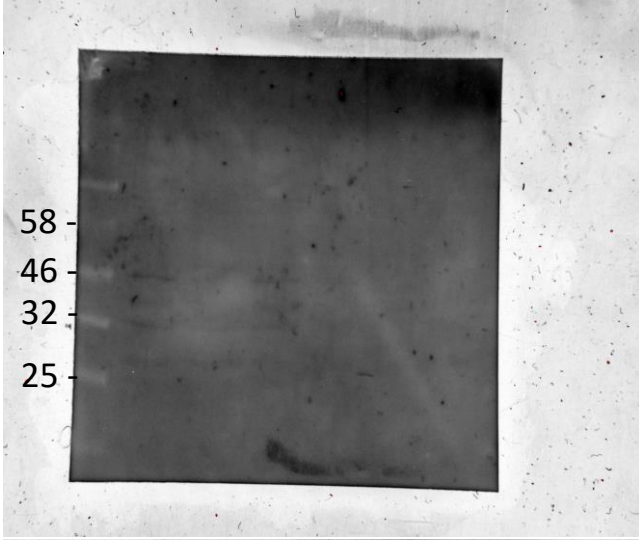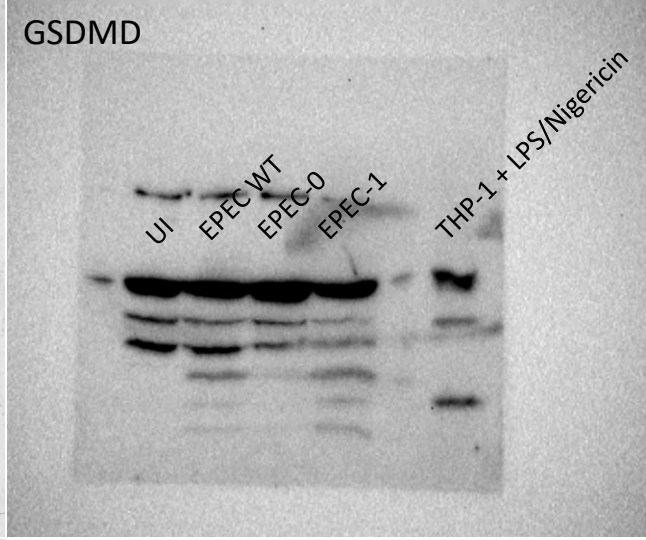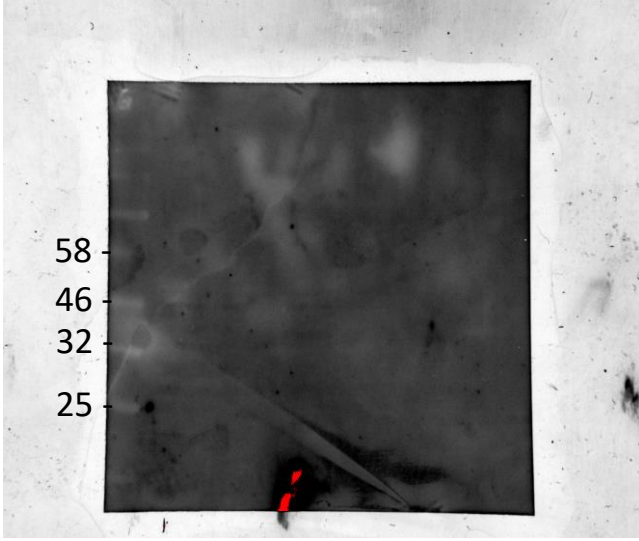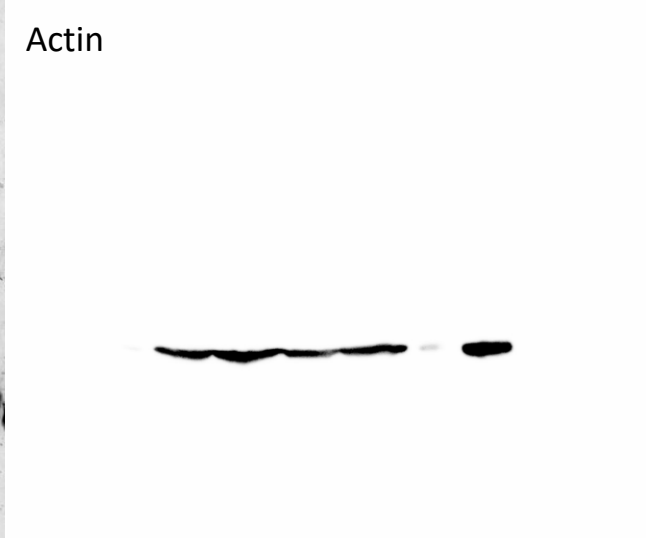

2J

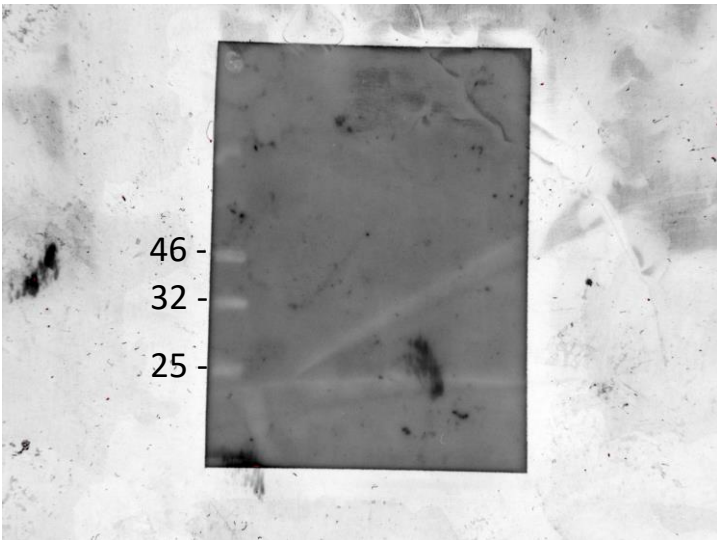

GSDMD

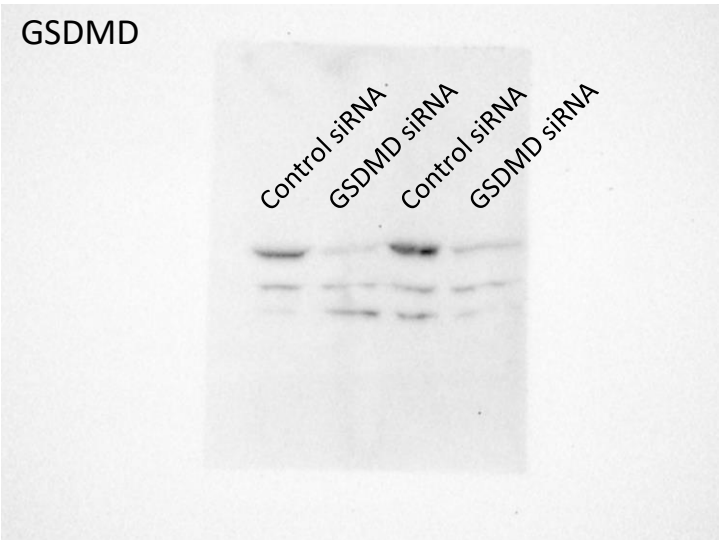

Actin

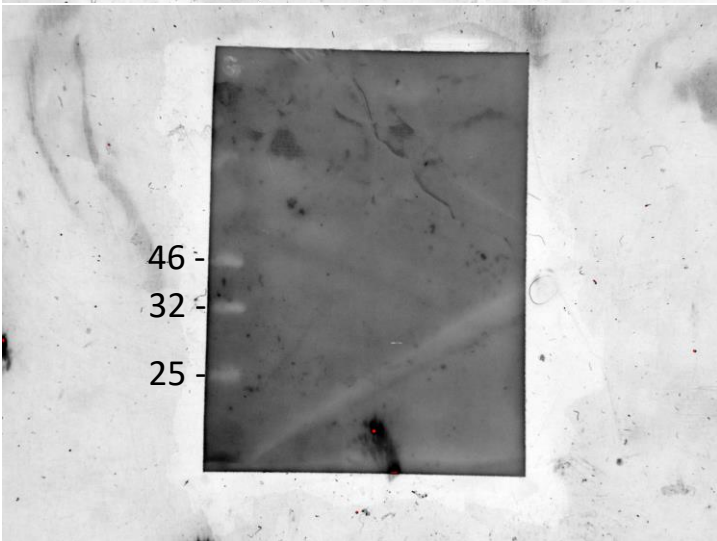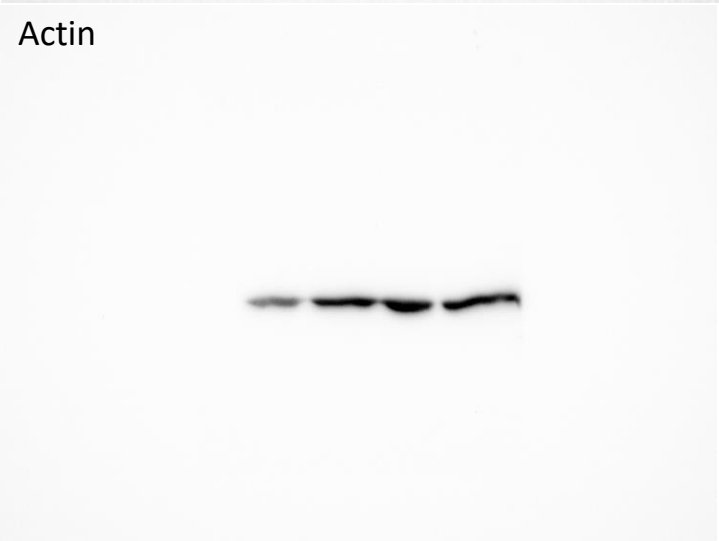

2K

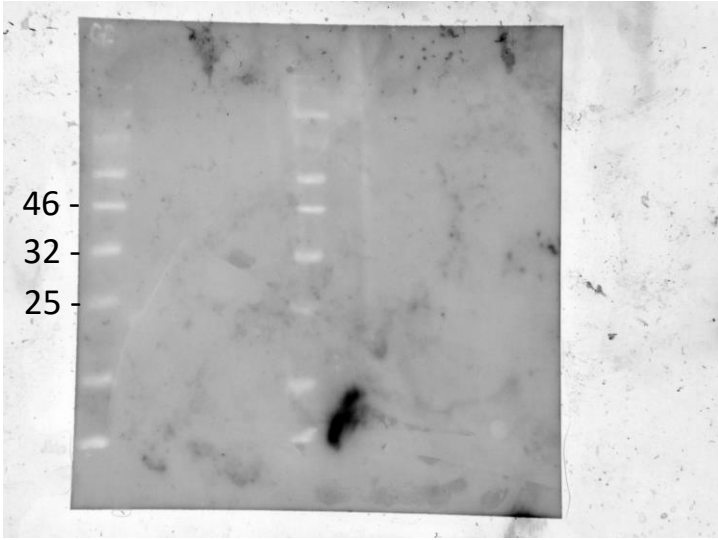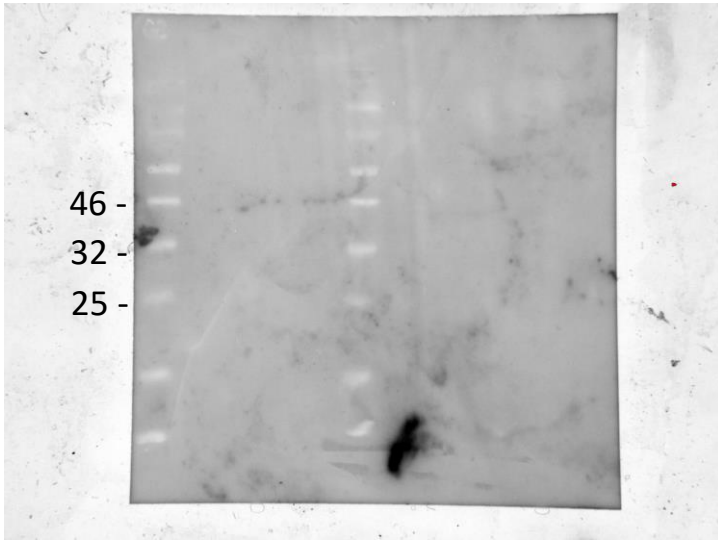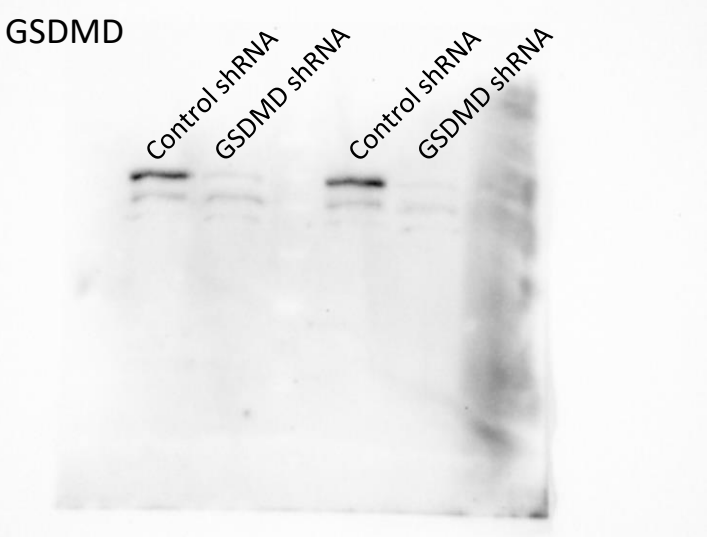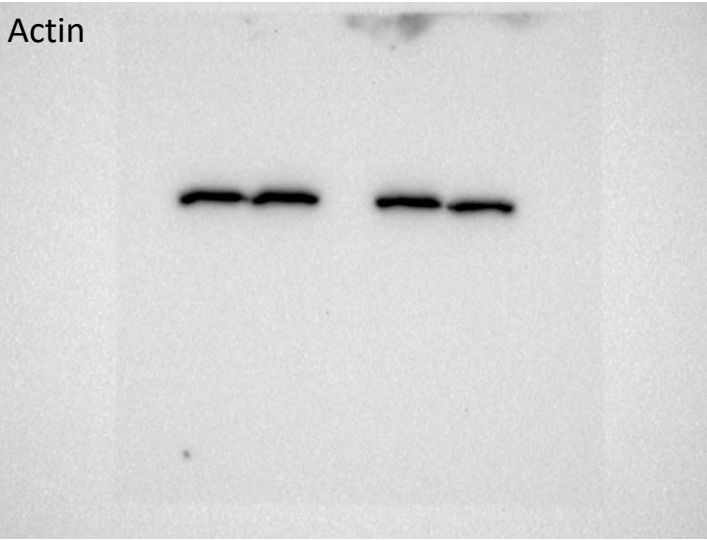

S2A

134 -  
100 -  
80 -

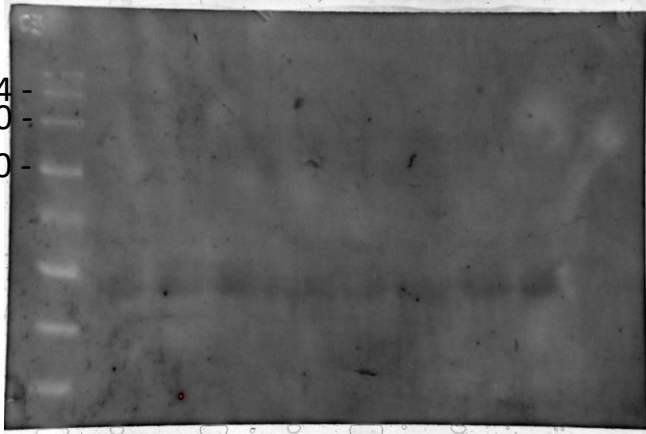

134 -  
100 -  
80 -

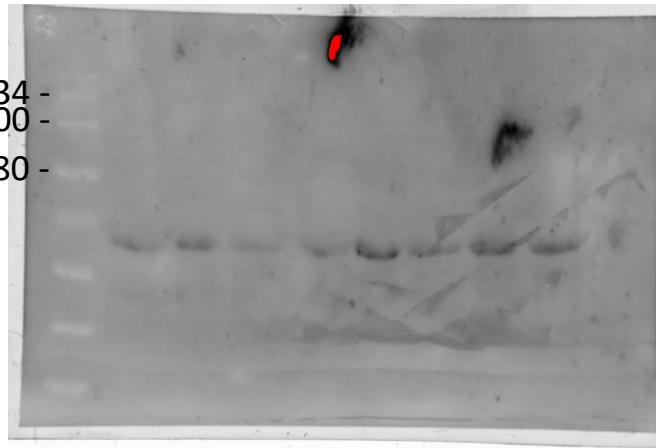

PARP1

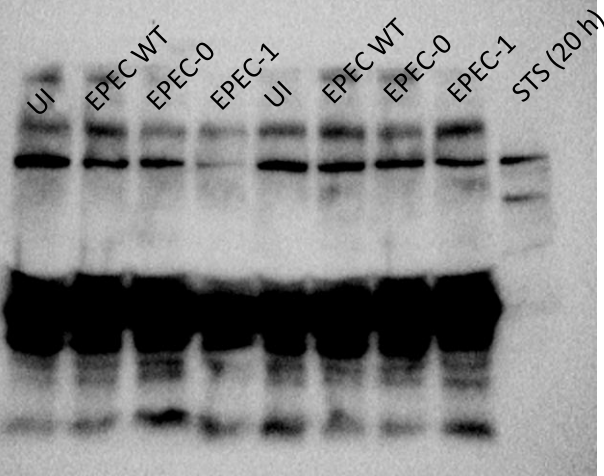

Actin

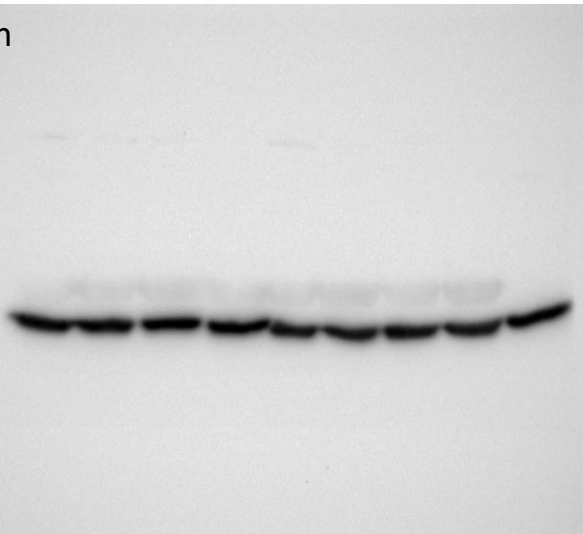

S2G

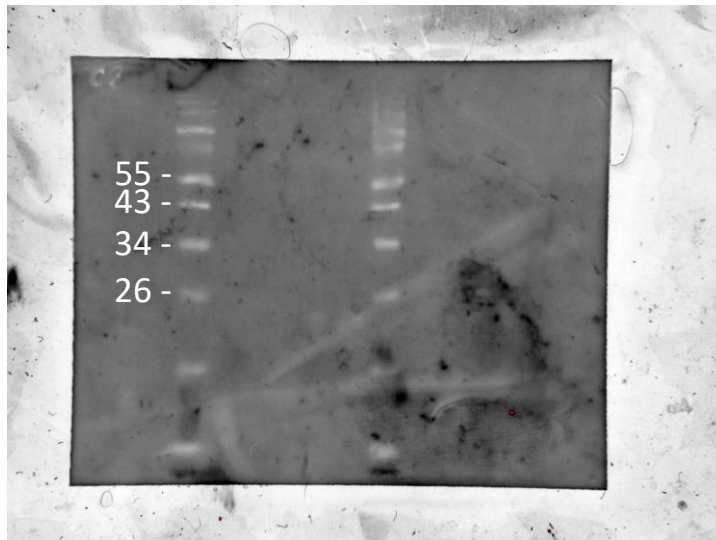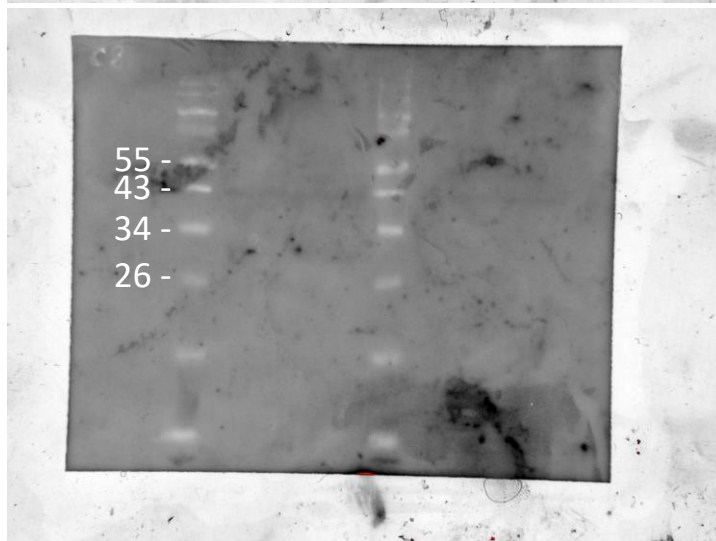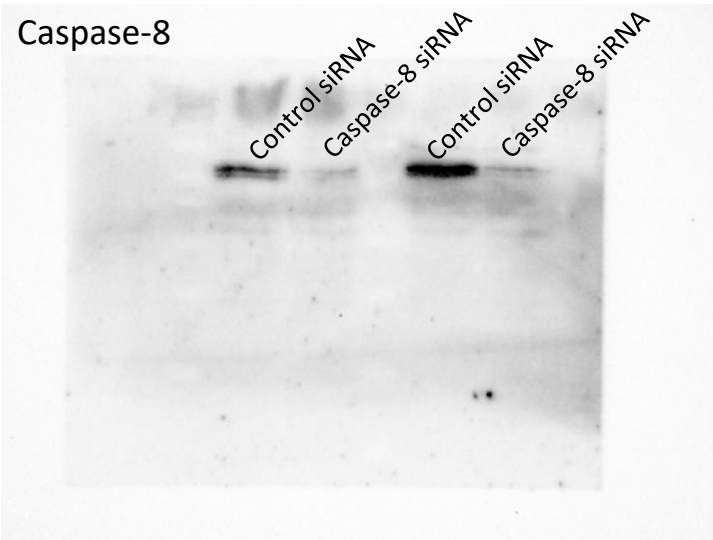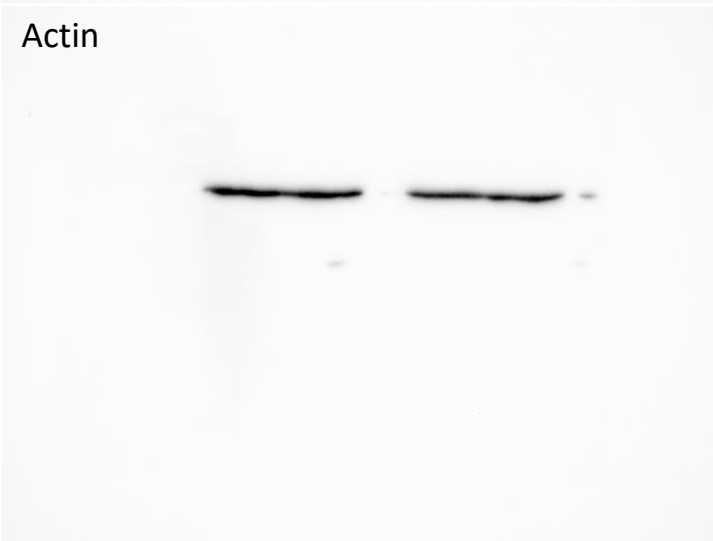

S2I

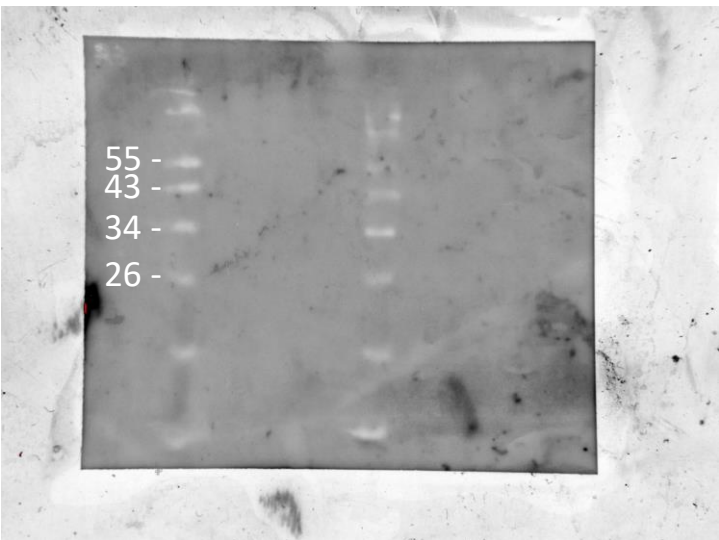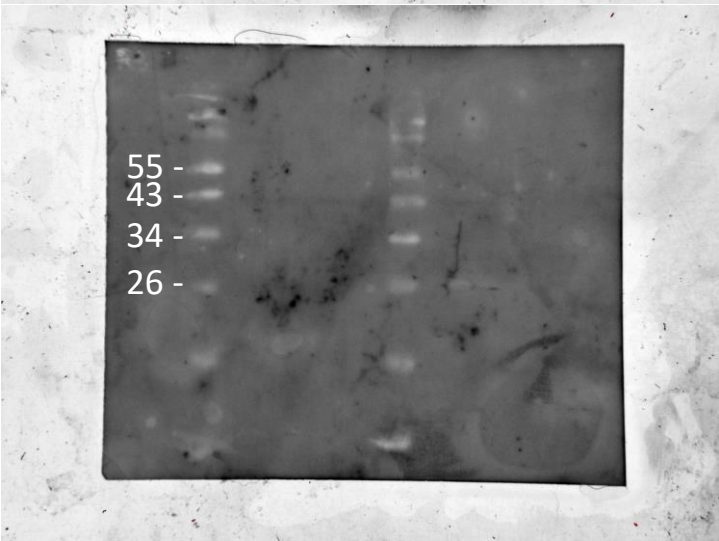

RIPK3

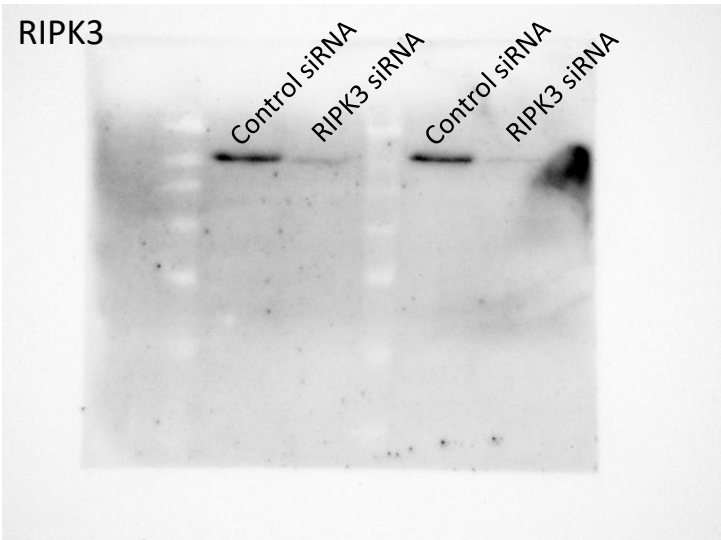

Actin

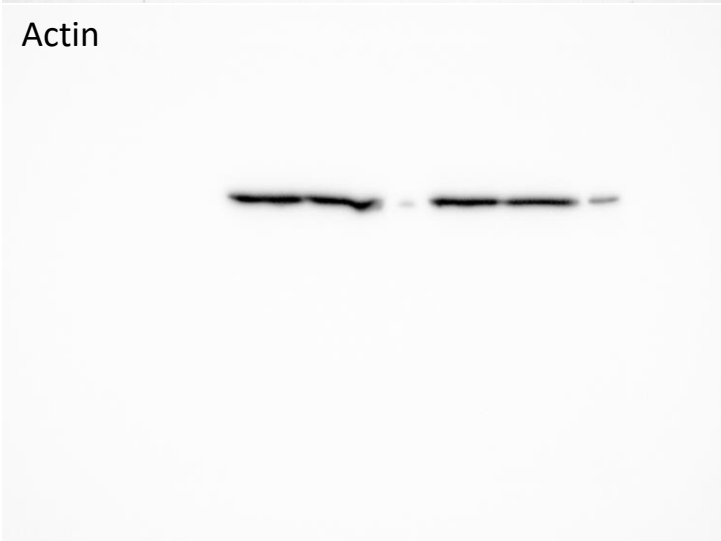

Supplement: S1 Raw Images — (PDF) [file pbio.3000986.s002.pdf]

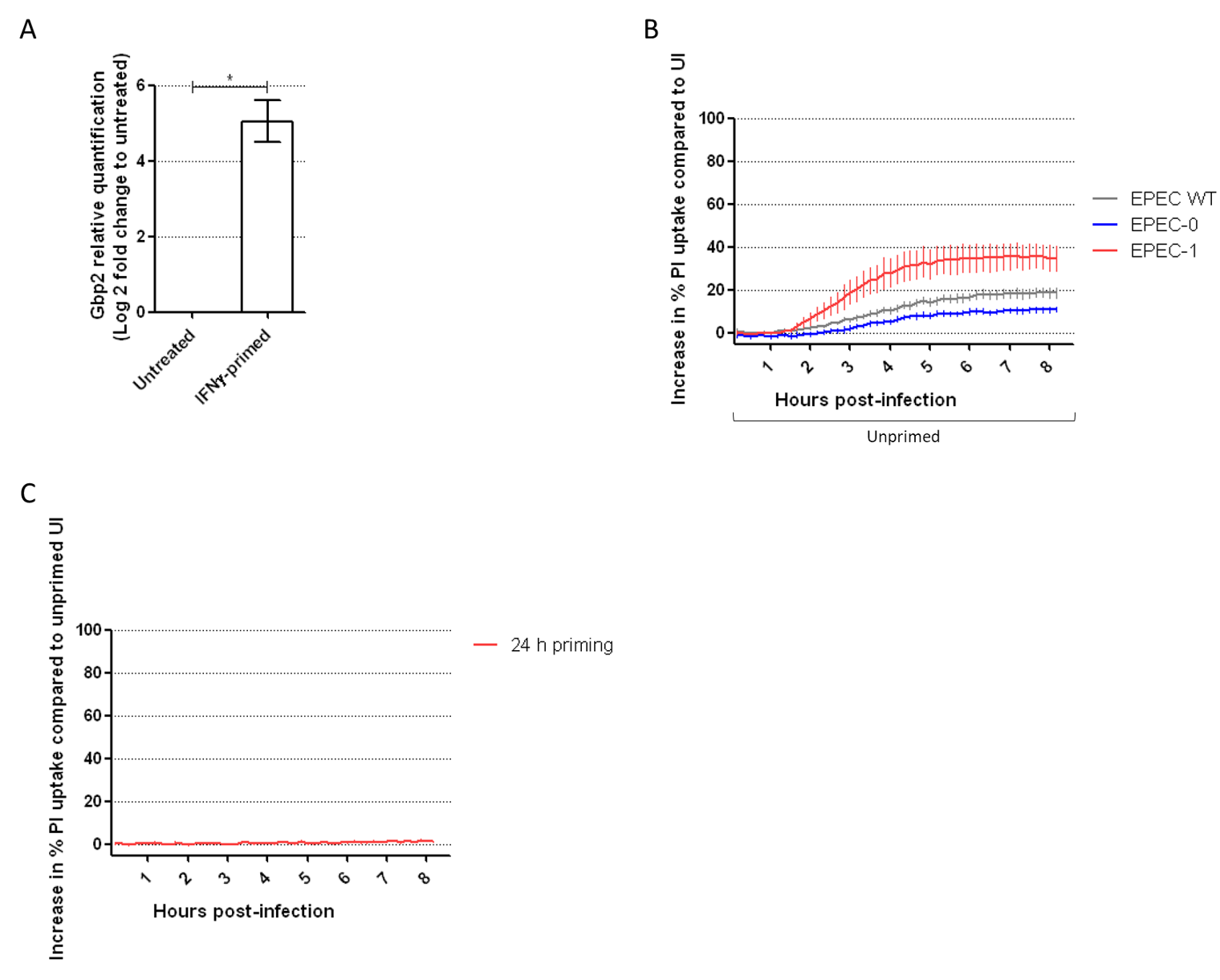

Supplement: S1 Fig — (A) Gbp2 expression level was measured by qRT-PCR in IFNγ-primed cells and normalised to the Gbp2 expression level in the untreated cells. Means ± SEM from n = 3 independent biological repeats. Statistical significance was determined using 2-tailed t test. * p ≤ 0.05. (B) PI uptake into unprimed SNU-C5 cells infected with EPEC WT, EPEC-0 and EPEC-1. Measurements were taken every 10 min. The time-course PI uptake was plotted. PI uptake results were normalised by UI. Means ± SEM from n = 5 independent biological repeats are shown. (C) PI uptake into IFNγ-primed uninfected cells. PI uptake results were normalised to unprimed UI cells. Means ± SEM from n = 7 independent biological repeats are shown. The underlying data for this figure can be found in S1 Data. EPEC, enteropathogenic Escherichia coli; IFNγ, interferon gamma; PI, propidium iodide; qRT-PCR, real-time quantitative PCR; SEM, standard error of the mean; Tir, translocated intimin receptor; UI, uninfected; WT, wild-type. (TIF) [file pbio.3000986.s003.tif]

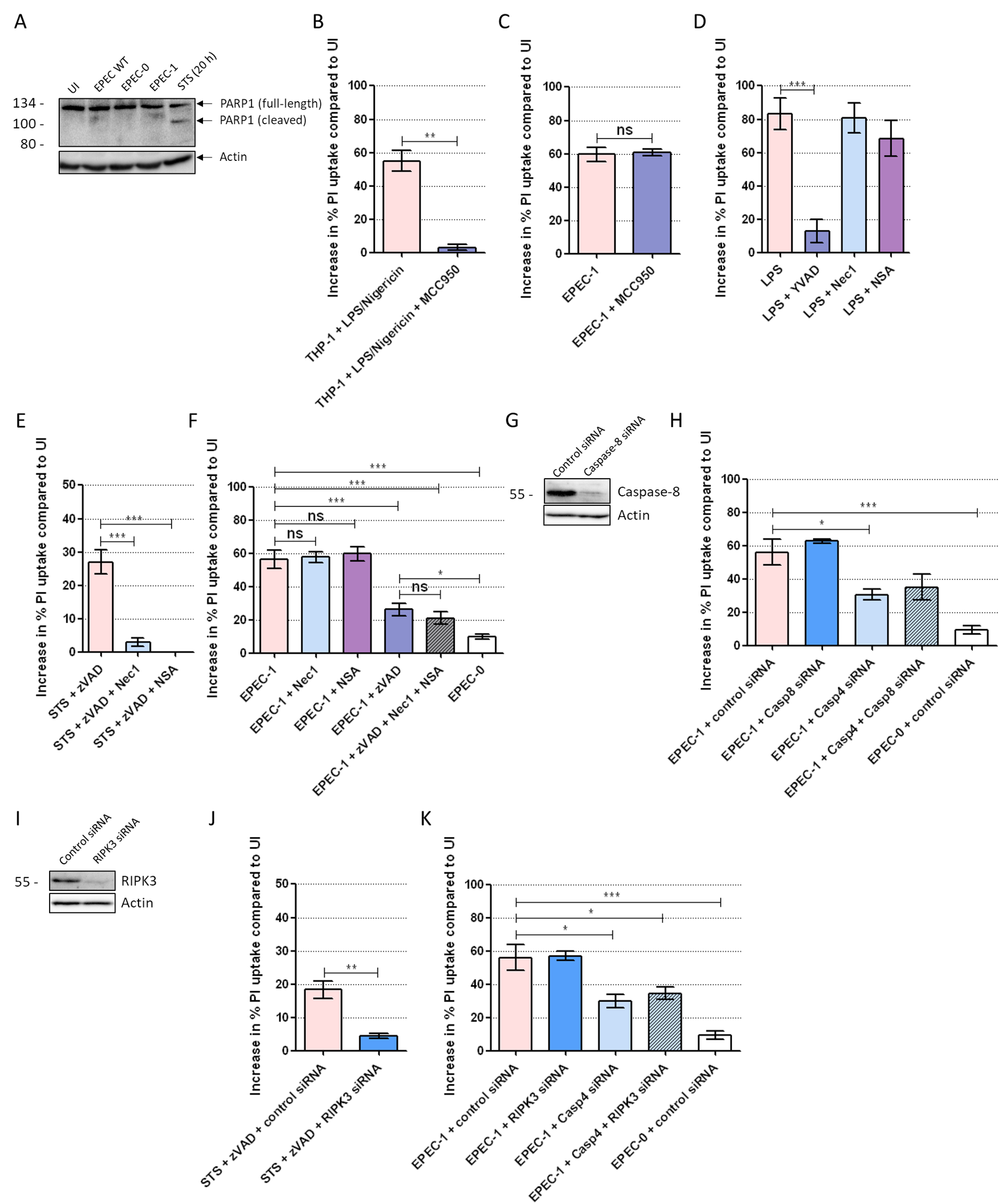

Supplement: S2 Fig — (A) Cell lysates of primed SNU-C5 cells infected with EPEC WT, EPEC-0, and EPEC-1, or treated by STS for 20 h, were used for PARP1 western blot. Representative blots were shown. (B) PI uptake into THP1 cells treated by LPS for 3 h followed by nigericin for 8 h, with or without MCC950 treatment 30 min before nigericin addition. Means ± SEM from n = 3 independent biological repeats. (C) PI uptake into primed SNU-C5 cells infected with EPEC-1 treated with MCC950 30 min before infection. Means ± SEM from n = 3 independent biological repeats. (D, E) PI uptake into SNU-C5 cells treated by YVAD, Nec1 and NSA 30 min before LPS transfection (D) or STS and zVAD treatment (E). Means ± SEM from n = 3 independent biological repeats. (F) PI uptake into SNU-C5 cells infected with EPEC-1 treated with Nec1, NSA, zVAD, and a combination of zVAD, Nec1, and NSA 30 min before infection. Means ± SEM from n = 3 independent biological repeats. (G) Caspase-8 western blot of SNU-C5 cells transfected with caspase-8 siRNA. Representative blot from n = 3 independent biological repeats are shown. (H) PI uptake into SNU-C5 cells transfected by caspase-4, GSDMD or caspase-8 siRNA or combinations of them and infected with EPEC-1. Means ± SEM from n = 3 independent biological repeats. (I) RIPK3 western blot of SNU-C5 cells transfected with RIPK3 siRNA. Representative blot from n = 3 independent biological repeats are shown. (J) PI uptake into SNU-C5 cells transfected by RIPK3 siRNA treated by STS and zVAD. Means ± SEM from n = 3 independent biological repeats. (K) PI uptake into SNU-C5 cells transfected by caspase-4, GSDMD or RIPK3 siRNA or combinations of them and infected with EPEC-1. Means ± SEM from n = 3 independent biological repeats. Statistical significance was determined using 2-tailed t test (B, C, J) and 1-way ANOVA with Tukey posttest (D, E, F, H, K). * p ≤ 0.05; ** p ≤ 0.01; *** p ≤ 0.001. The underlying data for this figure can be found in S1 Data. ANOVA, analysis of variance; EPEC, en [file pbio.3000986.s004.tif]

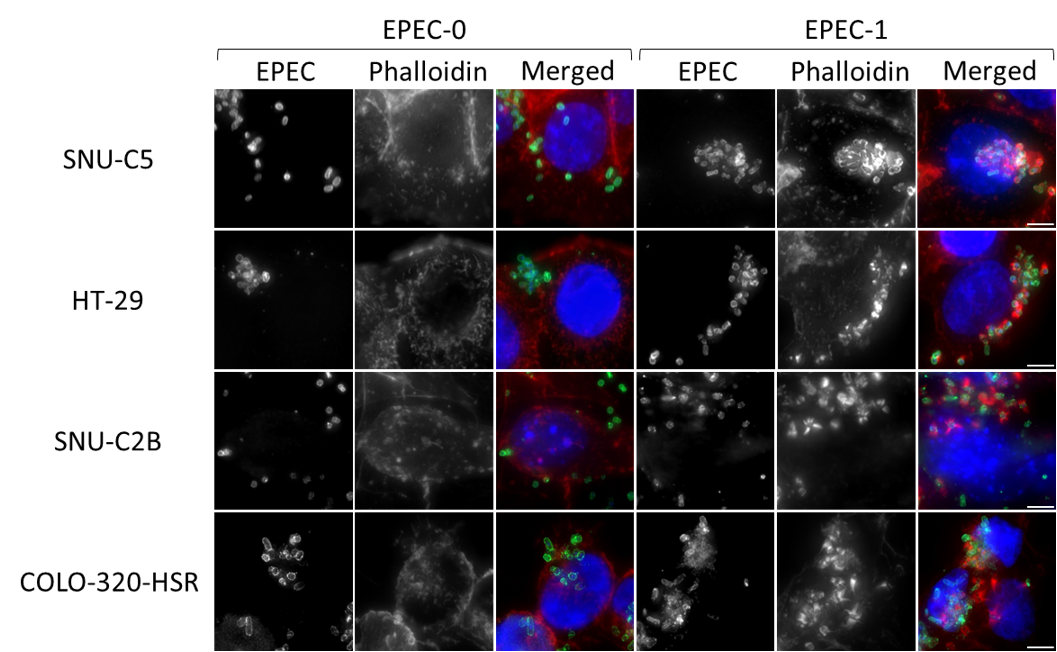

Supplement: S3 Fig — Immunofluorescence staining of SNU-C5, HT-29, SNU-C2B and COLO-320-HSR cells were infected with EPEC-0 and EPEC-1 for 4 h. DAPI: blue; EPEC: green; Phalloidin: red. Representative images from n = 3 independent biological repeats are shown. Error bar: 5 μm. The underlying data for this figure can be found in S1 Data. EPEC, enteropathogenic Escherichia coli; Tir, translocated intimin receptor. (TIF) [file pbio.3000986.s005.tif]

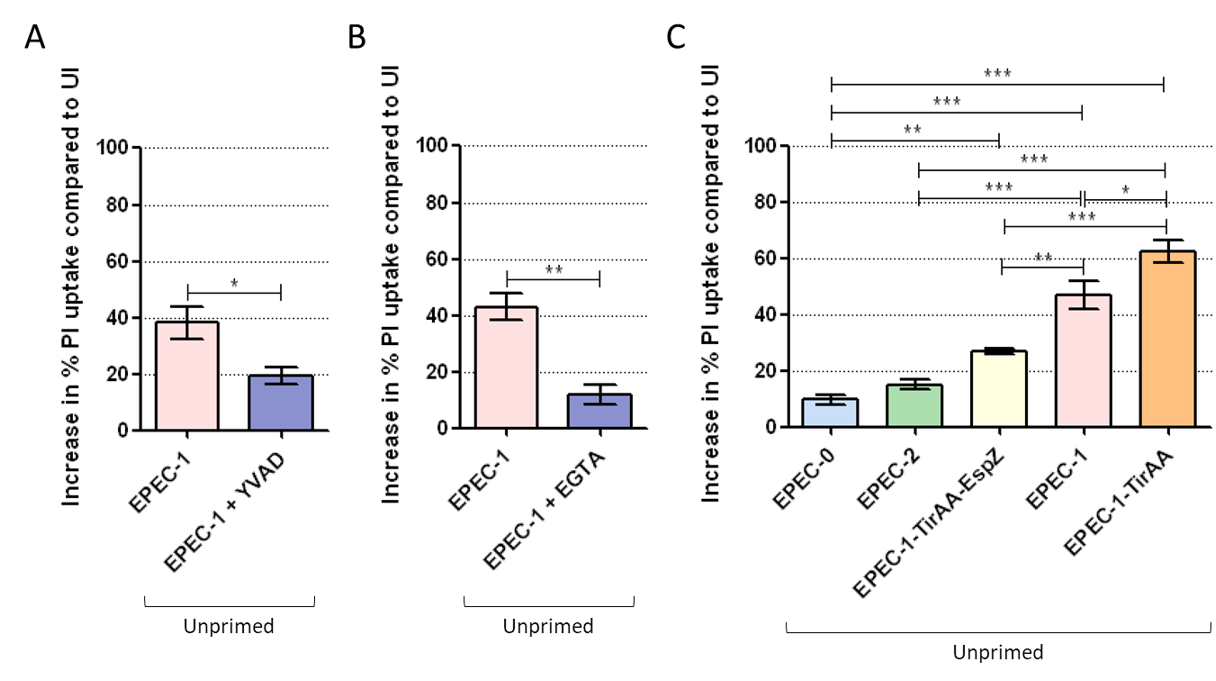

Supplement: S4 Fig — (A, B) PI uptake into unprimed SNU-C5 cells infected with EPEC-1 with 30 min pretreatment with YVAD (A) or EGTA (B). Means ± SEM from n = 3 independent biological repeats. (C) PI uptake into unprimed SNU-C5 cells infected with EPEC-0, EPEC-2, EPEC-1-TirAA-EspZ, EPEC-1 and EPEC-1-TirAA. Means ± SEM from n = 5 independent biological. Statistical significance was determined using 2-tailed t test (A, B) and 1-way ANOVA with Tukey posttest (C). * p ≤ 0.05; ** p ≤ 0.01; *** p ≤ 0.001. The underlying data for this figure can be found in S1 Data. ANOVA, analysis of variance; EPEC, enteropathogenic Escherichia coli; PI, propidium iodide; SEM, standard error of the mean;Tir, translocated intimin receptor; YVAD, z-YVAD-fmk. (TIF) [file pbio.3000986.s006.tif]

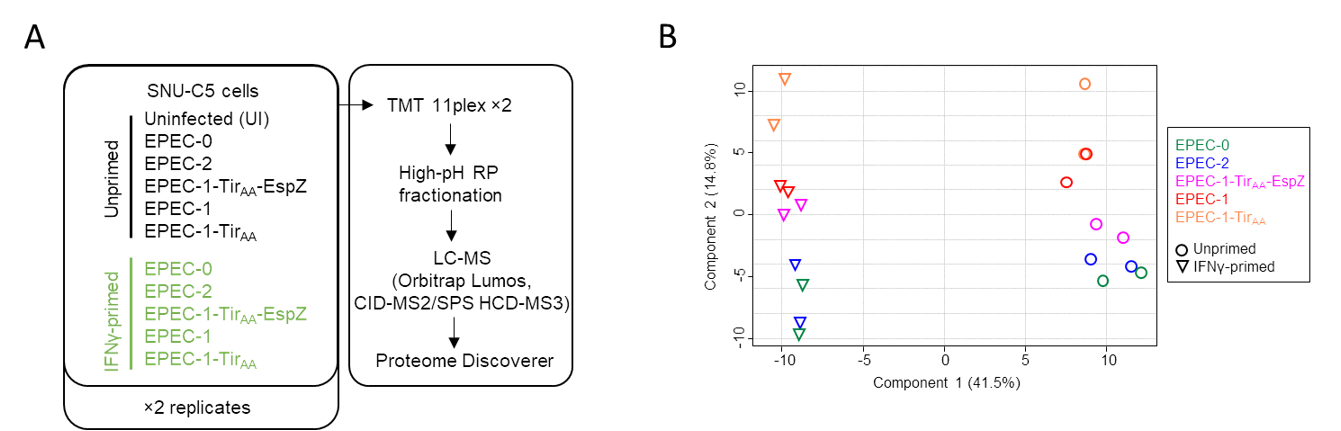

Supplement: S5 Fig — (A) IFNγ-primed and unprimed SNU-C5 cells were infected with the indicated EPEC strains for 2 h. Extracted proteins were digested with trypsin and peptides were labelled with the TMT 11plex reagents in 2 separate replicate batches. TMT-labelled peptides were subjected to offline high-pH reversed-phase fractionation followed by LC–MS analysis. (B) PCA of proteomics data. EPEC, enteropathogenic Escherichia coli; IFNγ, interferon gamma; LC–MS, liquid chromatography–mass spectrometry; PCA, principal component analysis; TMT, tandem mass tag. (TIF) [file pbio.3000986.s007.tif]

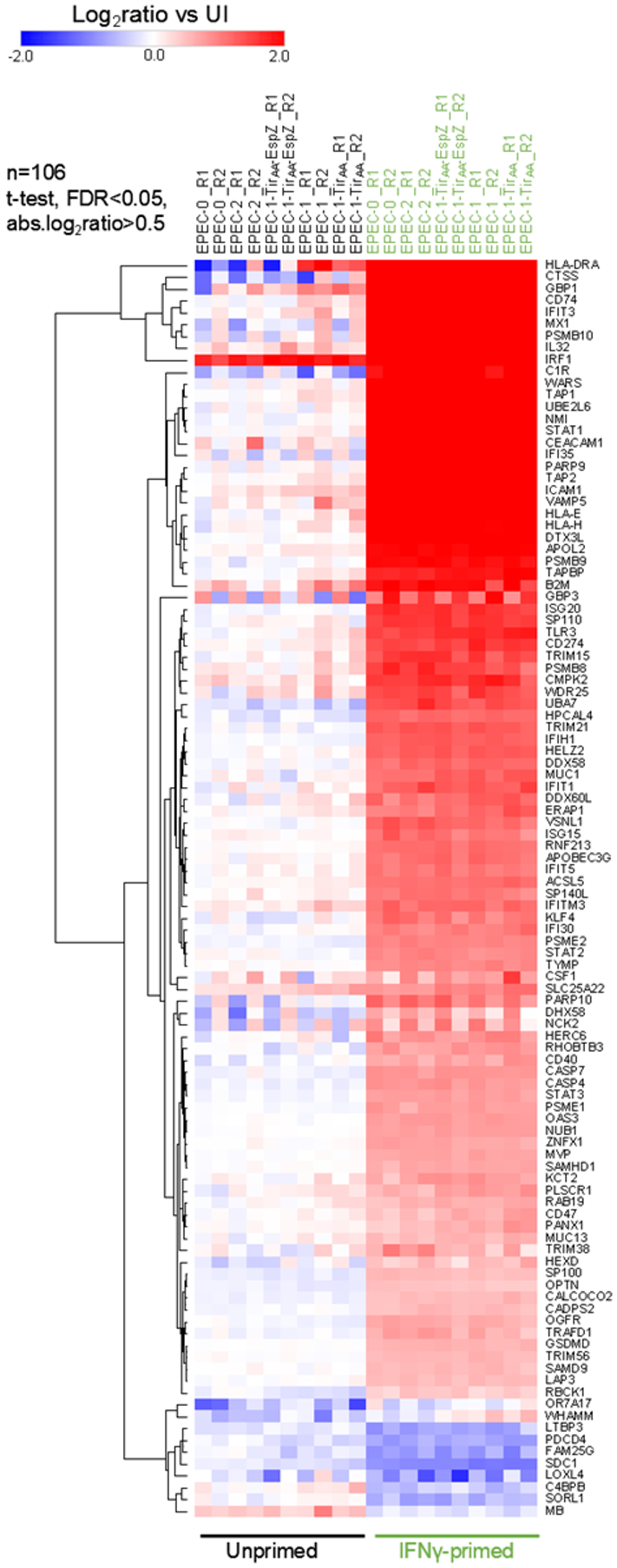

Supplement: S6 Fig — Heatmap of the differentially regulated proteins between IFNγ-primed and unprimed SNU-C5 cells (t test, FDR < 0.05, absolute log2 ratio versus uninfected > 0.5). The underlying data for this figure can be found in S1 Data. FDR, false discovery rate; IFNγ, interferon gamma. (TIF) [file pbio.3000986.s008.tif]

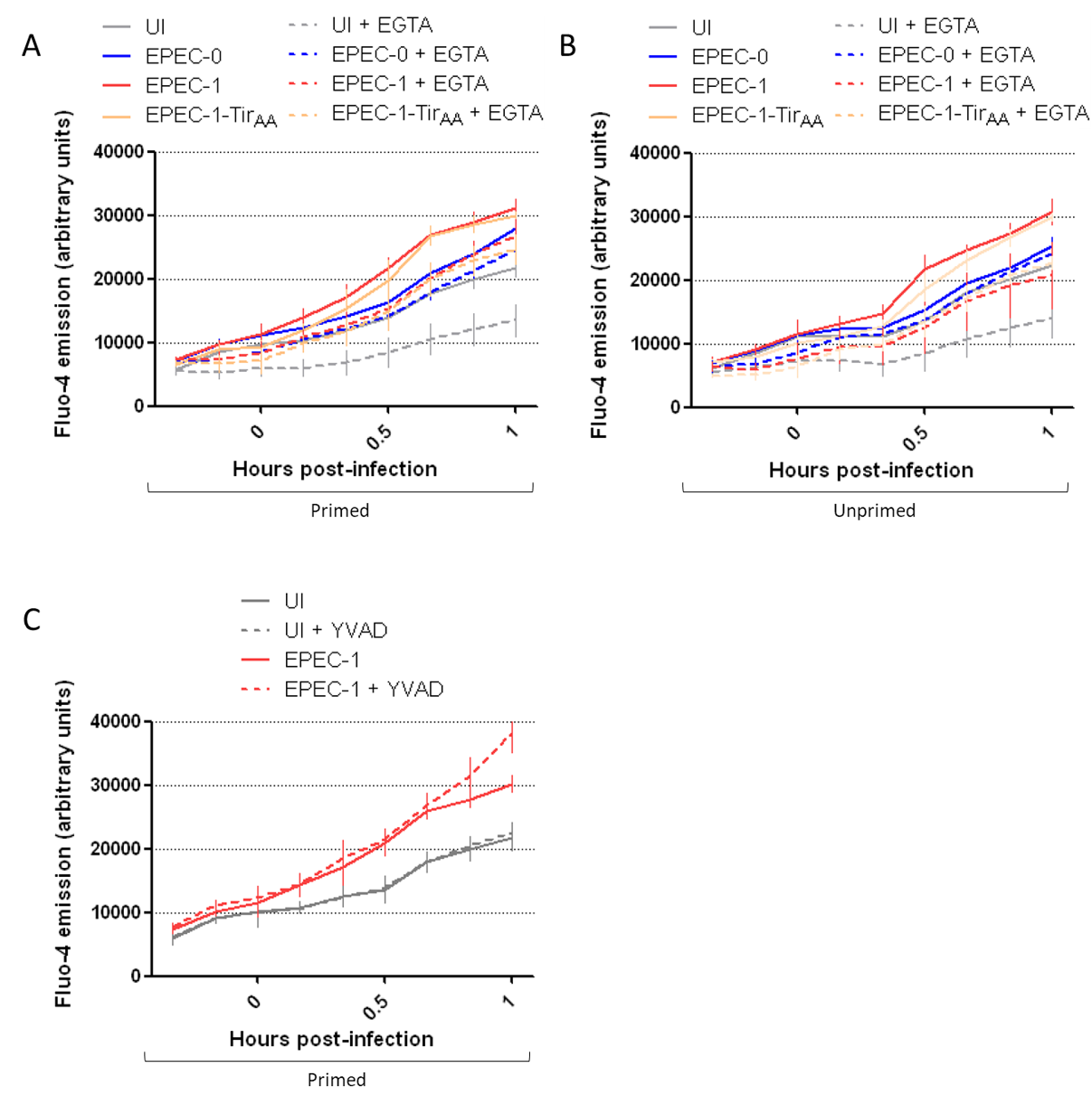

Supplement: S7 Fig — Fluo-4 assay performed on primed (A, C) and unprimed (B) SNU-C5 cells infected with EPEC-0 (A, B), EPEC-1 (A-C) and EPEC-1-TirAA (A, B) with or without 30 min pretreatment with EGTA (A, B) and YVAD (C). Means ± SEM from n = 3 independent biological repeats. The underlying data for this figure can be found in S1 Data. EPEC, enteropathogenic Escherichia coli; SEM, standard error of the mean; Tir, translocated intimin receptor; YVAD, z-YVAD-fmk. (TIF) [file pbio.3000986.s009.tif]

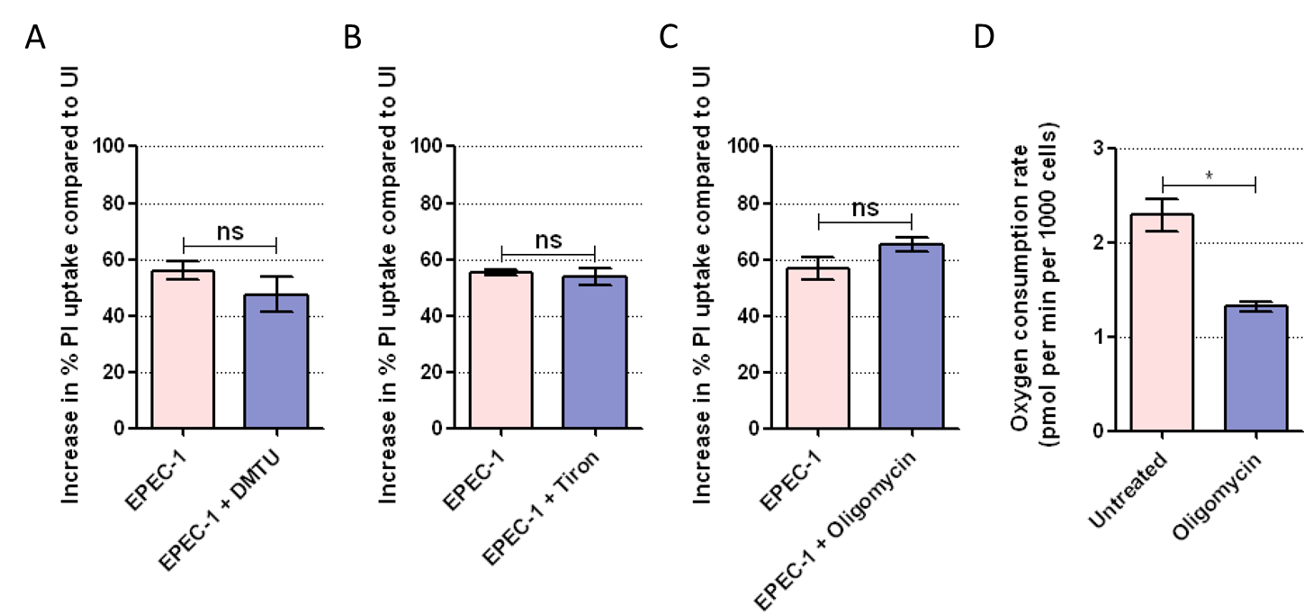

Supplement: S8 Fig — (A–C) PI uptake into primed SNU-C5 cells infected with EPEC-1, with 30 min pretreatment with DMTU (A), Tiron (B) or oligomycin (C). Means ± SEM from n = 3 independent biological. (D) OCR measurement was performed on SNU-C5 cells before or after oligomycin treatment. Means ± SEM from n = 3 independent biological repeats are shown. Statistical significance was determined using 2-tailed t test. ns, nonsignificant; * p ≤ 0.05; ** p ≤ 0.01; *** p ≤ 0.001. The underlying data for this figure can be found in S1 Data. DMTU, N,N′-dimethylthiourea; EPEC, enteropathogenic Escherichia coli; OCR, oxygen consumption rate; PI, propidium iodide; SEM, standard error of the mean. (TIF) [file pbio.3000986.s010.tif]
