# Supplementary material for: Clustering of Tir during enteropathogenic E. coli infection triggers calcium influx–dependent pyroptosis in intestinal epithelial cells
Source: PLoS Biol. 2020 Dec 30;18(12):e3000986. doi: 10.1371/journal.pbio.3000986 (PMC7773185; doi:10.1371/journal.pbio.3000986)
Supplement: S1 Table — (A) Bacterial strains. (B) Plasmids. (C) Primer sequences. (D) siRNA and miRNA30E sequences. (E) Immunofluorescence antibodies and reagents. (F) Western blot antibodies. siRNA, small interfering RNA. (DOCX) [file pbio.3000986.s011.docx]

**Table A. Bacterial strains**

| **Bacterial strains** | **Description/Plasmids** | **Reference** |
| --- | --- | --- |
| EPEC WT | Wild-type E2348/69 | (1) |
| EPEC-0 | Effectorless | (2) |
| EPEC-1 | EPEC-0 with Tir expression | (2) |
| EPEC-1-Tir_AA_ | EPEC-1 with Tir Y454A and Y474A mutations | This study |
| EPEC-1-Tir_AA_-EspZ | pSA10-*espZ* | This study |
| EPEC-2 | EPEC-0 with Tir and EspZ expression | (2) |
| EPEC-1Δ*eae* | EPEC-1 with Intimin deletion | (2) |
| EPEC-1-NleF | pACYC-*nleF* | This study |
| EPEC-1-Tir_AA_-NleF | pACYC-*nleF* | This study |
| EPEC Δ*tir* | EPEC WT with Tir deletion | (3) |
| *E. coli* CC118λpir | pSEVA plasmid propagating strain | (4) |
| *E. coli* CC118λpir pSEVA-*tir* | pSEVA-*tir* | This study |
| *E. coli* CC118λpir pSEVA-*tir_AA_* | pSEVA-*tir_AA_* | This study |
| *E. coli* 1047 pRK2013 | pSEVA plasmid conjugation helper strain | (5) |

**Table B. Plasmids**

| **Plasmids** | **Description** | **Resistance** | **Reference** |
| --- | --- | --- | --- |
| pSEVA612S | R6K ori, oriT, I-*Sce*I restriction sites flanking multicloning site | Gm | (6) |
| pSEVA612S-*tir* | pSEVA612S plasmid encoding Tir | Gm | This study |
| pSEVA612S-*tir_AA_* | pSEVA612S plasmid encoding Tir_AA_ | Gm | This study |
| pACBSR | p15A ori, P_BAD_ promoter, I-*Sce*I endonuclease and λ Red genes | Sm | (7) |
| pSA10-*espZ* | pSA10 plasmid encoding EspZ | Am | (8) |
| pACYC-*nleF* | pACYC plasmid encoding EPEC NleF | Cm | (9) |

Gm: Gentamicin; Sm: Streptomycin; Am: Ampicillin; Cm: Chloramphenicol.

**Table C. Primer sequences**

| **Primers** | **Sequences** |
| --- | --- |
| 1.1.1 Fwd HindIII-HR1-Tir | CGCGAAGCTTGGGGAAACTTACTGCGCTGTTATTTTTTTTC |
| 1.2.1 Rvs BamHI-HR2-Tir | CGCGGGATCCGTTTGGGCTCCACCACAATGAG |
| 1.3.2 Rvs Y454A | CGATAGACTATTCCGAGCCCCCCCAACTTCAGCAGCTGGATTAACCAC |
| 1.4.2 Fwd Y474A | GCTCATCAGCCAGAAGAGCATATTGCTGATGAGGTCGCTGCAGATCCTG |
| 1.5 Fwd check Tir | CGTTTTATTTTTATAGGTG |
| 1.6 Rvs check Tir | CAGCACTAAGCATAATAAATG |
| 1.7 204 Fwd seq TirAA | GGTGTTGGCAGCGCTTC |
| 1.8 352 Rvs seq TirAA | CATGCTGATCCTCATATC |
| M13-FP | TGTAAAACGACGGCCAGT |
| M13-RP | CAGGAAACAGCTATGACC |
| hGBP2-Fwd | GTCTCACACCAAGGGAATCTG |
| hGBP2-Rvs | AAGGCAAAGATCCAGGAGTC |
| hGAPDH-Fwd | TCGACAGTCAGCCGCATCTTCTTT |
| hGAPDH-Rvs | ACCAAATCCGTTGACTCCGACCTT |

**Table D. siRNA sequences**

| **siRNA** | **Sequences** |
| --- | --- |
| L-004404-05 Caspase-4 | GGACUAUAGUGUAGAUGUA |
| L-004404-06 Caspase-4 | CAACGUAUGGCAGGACAAA |
| L-004404-07 Caspase-4 | GAACUGUGCAUGAUGAGAA |
| L-004404-08 Caspase-4 | UAACAUAGACCAAAUAUCC |
| J-016207-05 GSDMD | CCACGUGCUUGCAGGGUGA |
| J-016207-06 GSDMD | GUCCUUCUCUUCCCGGAUA |
| J-016207-07 GSDMD | GCACCUCAAUGAAUGUGUA |
| J-016207-08 GSDMD | GGAACUCGCUAUCCCUGUU |
| D-001810-01 Non-targeting | UGGUUUACAUGUCGACUAA |
| D-001810-02 Non-targeting | UGGUUUACAUGUUGUGUGA |
| D-001810-03 Non-targeting | UGGUUUACAUGUUUUCUGA |
| D-001810-04 Non-targeting | UGGUUUACAUGUUUUCCUA |

**Table E. Immunofluorescence antibodies and reagents**

| **Antibodies/reagents** | **Species** | **Dilutions** | **Catalogue** | **Sources** |
| --- | --- | --- | --- | --- |
| **Primary antibodies & dyes** | | | | |
| Anti-EPEC O127:H6 | Rabbit | 1: 200 | N/A (serum) | VLA |
| Anti-p65 | Rabbit | 1: 200 | sc-109 | Santa Cruz Biotechnology |
| **Secondary antibodies & dyes** | | | | |
| DAPI | N/A | 1: 1,000 | D3571 | Sigma |
| Phalloidin-Alexa 647 | N/A | 1: 200 | 23127-AAT | Stratech |
| Anti-rabbit IgG-Alexa 488 | Donkey | 1: 200 | N/A | Jackson Immunoresearch |

**Table F. Western blot antibodies**

| **Antibodies/reagents** | **Species** | **Dilutions** | **Catalogue** | **Sources** |
| --- | --- | --- | --- | --- |
| **Primary antibodies** | | | | |
| Anti-Casp4 | Mouse | 1: 500 (5% milk/PBST) | Sc-56056 | Santa Cruz Biotechnology |
| Anti-GSDMD | Rabbit | 1: 1,000 (5% BSA/TBST) | 96458S | Cell Signaling Technology |
| Anti-PARP1 | Rabbit | 1: 1,000 (5% milk/PBST) | 9542 | Cell Signaling Technology |
| **Secondary antibodies** | | | | |
| Anti-rabbit HRP | Goat | 1: 10,000 (5% milk/PBST) | 111-035-008 | Jackson Immunoresearch |
| Anti-mouse HRP | Goat | 1: 10,000 (5% milk/PBST) | 115-035-008 | Jackson Immunoresearch |
| Anti-β-actin HRP | Mouse | 1: 100,000 (5% milk/PBST) | A3854 | Sigma |

**Reference**

1. Levine M, Bergquist E, Nalin D, Waterman D, Hornick R, Young C, et al. *Escherichia coli* strains that cause diarrhoea but do not produce heat-labile or heat-stable enterotoxins and are non-invasive. Lancet. 1978;1(8074):1119–22.

2. Cepeda-Molero M, Berger CN, Walsham ADS, Ellis SJ, Wemyss-Holden S, Schuller S, et al. Attaching and effacing (A/E) lesion formation by enteropathogenic *E. coli* on human intestinal mucosa is dependent on non-LEE effectors. PLoS Pathog. 2017;1–23.

3. Berger CN, Crepin VF, Jepson MA, Arbeloa A, Frankel G. The mechanisms used by enteropathogenic *Escherichia coli* to control filopodia dynamics. Cell Microbiol. 2009;11(2):309–22.

4. Herrero M, Lorenzo VDE, Timmis KN. Transposon vectors containing non-antibiotic resistance selection markers for cloning and stable chromosomal insertion of foreign genes in Gram-negative bacteria. J Bacteriol. 1990;172(11):6557–67.

5. Figurski DH, Helinski DR. Replication of an origin-containing derivative of plasmid RK2 dependent on a plasmid function provided in trans. PNAS. 1979;76(4):1648–52.

6. Martínez-García E, Aparicio T, Goñi-Moreno A, Fraile S, de Lorenzo V. SEVA 2.0: an update of the Standard European Vector Architecture for de-/re-construction of bacterial functionalities. Nucleic Acids Res. 2015;43:1183–9.

7. Ruano-Gallego D, Álvarez B, Luis A. Engineering the controlled assembly of filamentous injectisomes in *E. coli* K‑12 for protein translocation into mammalian cells. ACS Synth Biol. 2015;4:1030–41.

8. Berger CN, Crepin VF, Baruch K, Mousnier A, Rosenshine I, Frankel G. EspZ of enteropathogenic and enterohemorrhagic *Escherichia coli* regulates type III secretion system protein translocation. MBio. 2012;3(5):1–12.

9. Pallett MA, Crepin VF, Serafini N, Habibzay M, Kotik O, Sanchez-Garrido J, et al. Bacterial virulence factor inhibits caspase-4/11 activation in intestinal epithelial cells. Mucosal Immunol. 2017;10(3):602–12.
